# Equitable inclusion of people with disabilities in clinical trials: a scoping review

**DOI:** 10.1136/bmjopen-2025-108550

**Published:** 2026-02-04

**Authors:** Grace Jo, Franz Castro, Caroline Cerilli, Varshini Varadaraj, Lori Rosman, Kaloyan Kamenov, Darryl Barrett, Bonnielin Swenor

**Affiliations:** 1 Disability Health Research Center, Johns Hopkins University, Baltimore, Maryland, USA; 2 Welch Medical Library, Johns Hopkins University School of Medicine, Baltimore, Maryland, USA; 3 Disability Programme, World Health Organization, Geneva, Switzerland

**Keywords:** Health policy, Clinical Trial, PUBLIC HEALTH

## Abstract

**Abstract:**

**Objectives:**

People with disabilities are underrepresented in clinical trials, which impacts generalisability and ethical integrity of results. Despite international mandates to diversify participants, there is a lack of guidance on how to make trials disability-inclusive. This scoping review identifies and synthesises guidance, practices and recommendations supporting the inclusion of people with disabilities in clinical trials.

**Design:**

We conducted a scoping review following the Arksey and O'Malley framework and reported according to Preferred Reporting Items for Systematic Reviews and Meta-Analyses for scoping reviews.

**Data sources:**

Medline (OVID) and Public Affairs Information System Index (October 2024) searches identified peer-reviewed articles. Grey literature was identified through targeted advanced Google searches and website reviews (February–March 2025). Google Scholar was searched to identify recently published documents between March and April 2025.

**Eligibility criteria for selecting studies:**

Documents with guidance or policies related to inclusion of people with disabilities in clinical trials published in English from 2019 to 2025 were included.

**Data extraction and synthesis:**

Two independent reviewers screened titles, abstracts and full texts and extracted data, with adjudication by a third reviewer.

**Results:**

A total of 69 documents met inclusion criteria. Thematic synthesis identified eight recommendation domains: (1) inclusive and universal trial design, (2) accessible recruitment, (3) inclusive data collection, (4) equitable data analysis, (5) accessible reporting and dissemination, (6) ethical oversight and institutional review board inclusion, (7) supported and accessible consent processes and (8) the inclusion of people with disabilities as researchers and stakeholders. Themes cutting across all domains included guidance emphasising universal design, anti-ableist frameworks, supported decision-making, flexible protocols and engagement with disability communities.

**Conclusion:**

Disability-inclusive clinical trials are essential to ensure the validity of clinical trial results, uphold ethical responsibilities and advance health equity. While emerging frameworks offer direction on how to include people with disabilities in trials, current implementation remains inconsistent and largely voluntary. Clear, enforceable standards are needed to support governments, ethics boards, institutions and funders in making clinical trials more disability-inclusive.

**STRENGTHS AND LIMITATIONS OF THIS STUDY**

- This scoping review was not limited to a specific disability type or definition and contained the inclusion of peer-reviewed and grey literature.
- This review followed well-established guidelines for scoping reviews and adhered to reporting according to Preferred Reporting Items for Systematic Reviews and Meta-Analyses for scoping reviews.
- Limitations of this review include restriction to English-language documents, exclusion of unpublished or non-written resources, and smaller number of peer-reviewed databases searched due to rapid review timeframes.

## Introduction

 According to the WHO, there are over 1.3 billion people with disabilities worldwide.^1^ Despite this, people with disabilities are persistently underrepresented in clinical research, including clinical trials.^1^,^6^ While efforts to diversify clinical trial participation have intensified since the COVID-19 pandemic, the inclusion of people with disabilities has lagged.

However, the imperative to include people with disabilities in trials was reinforced by the 2022 World Health Assembly resolution (WHA 75.8), *Strengthening Clinical Trials to Provide High-Quality Evidence on Health Interventions and to Improve Research Quality and Coordination*.^7^ This resolution not only urges member states to ensure trials are health-needs driven, well designed and guided by principles of fairness, equity, justice and autonomy but also to include diverse populations in all steps of the trial process.^7^ The WHA 75.8 specifically calls on member states to ‘ensure trials are designed to reflect the heterogeneity of those who will ultimately use or benefit from the intervention being evaluated, and are conducted in diverse settings, including all major population groups the intervention is intended to benefit, with a particular focus on under-represented populations’.^7^

Despite such commitments, the participation of people with disabilities in trials is often hindered by a multitude of barriers. These barriers can occur across all phases and stages of trials, including a lack of budgeting for accommodations, inaccessible study designs and environments, and mistrust of research due to past unethical research practices (ie, experimentation on people without consent).^8^,^11^ Exclusionary practices in trials present the biggest barriers for people with disabilities. For instance, trials often rely on incorrect assumptions that people with disabilities are inherently unable to participate in trials due to their impairments. This misconception leads to systematic exclusion from trials, either explicitly through exclusionary designs and eligibility criteria or implicitly through inaccessible study designs and procedures.^8^,^12^ The broad diversity in the population (with various disability types including people with intellectual and developmental disabilities (IDD), mobility disabilities, sensory disabilities, mental health conditions and cognitive impairments) and the variance in defining disability can exacerbate these mistaken assumptions. In reality, most people with disabilities are fully capable of participating in research, particularly when reasonable accommodations are made. Their exclusion is often not based on actual limitations to participate in trials and is often not scientifically justified.^12^

Knowledge about the many barriers to inclusion in trials underscores a need for guidance on how to design disability-inclusive trials. Currently, guidance on how to include people with disabilities in clinical trials is limited, and the available guidance has not been assembled to ensure inclusion across all stages of the clinical trial process. To fill this knowledge gap, we conducted a rapid scoping review to examine the extent and nature of policies, practices and guidance available for the inclusion of people with disabilities in clinical trials globally. The goal of this work is to synthesise the evidence to offer a practical reference to support the integration of disability inclusion and equity into the design and conduct of clinical trials.

## Methods

### Search strategy

This scoping review was conducted following guidance from the Arksey and O’Malley framework and reported according to the Preferred Reporting Items for Systematic Reviews and Meta-Analyses for scoping reviews (PRISMA-ScR).^13 14^ A search strategy was developed with consultation with a librarian to address our research aim of identifying policies and practices of disability inclusion and exclusion in clinical trials. We followed the Population/Concept/Context framework to develop a search query for each search concept using a combination of MeSH and keyword terms (online supplemental tables 1 and 2). Our population of interest was people with disabilities; the concept was disability inclusion and exclusion policies, practices and guidance, and the context for this review was clinical research. Two databases (Medline OVID and Public Affairs Information Service (PAIS) Index) were searched and imported to Covidence on 18 October 2024. The protocol for this scoping review was not registered. We also conducted a grey literature search using customised Advanced Google searches from 25 February 2025 to 4 March 2025, to identify non-peer reviewed documents relevant to our research question. On 27 February 2025, we also used targeted website searches of relevant disability health agencies, organisations and advocate websites to provide a more specific approach, in addition to a set of broader Advanced Google searches. We conducted a final Google Scholar search on 15 April 2025, to collect any studies that were published after our initial search.

### Inclusion/exclusion criteria

Publications from January 2019 to April 2025 were included in this review. We only included publications in English and did not restrict documents based on disability type or location of publication. This project was a subset of a larger rapid scoping review focused on identifying guidance on the inclusion of people with disabilities in biomedical, health services and public health research. We included written documents providing data on policies, recommendations, guidance and practices of disability inclusion and exclusion in clinical research. Documents were not limited to a specific definition of disability to capture an overview of current clinical guidance across diverse disability definitions and types. Advanced Google and Google Scholar searches were used to identify publicly available information and documents including, but not limited to, press notes, organisational briefs, blog posts, institutional reports, guidelines, book chapters and workshop notes which focused on disability inclusion/exclusion in clinical research.

We excluded audio/visual content such as webinars and interviews; however, if a webinar or interview provided a written transcript, we included the transcript. Any documents not published in English or published prior to 2019 were excluded. Publications which did not provide guidance or policies on disability inclusion/exclusion in clinical research were excluded from this review.

### Search selection

**Figure 1:**
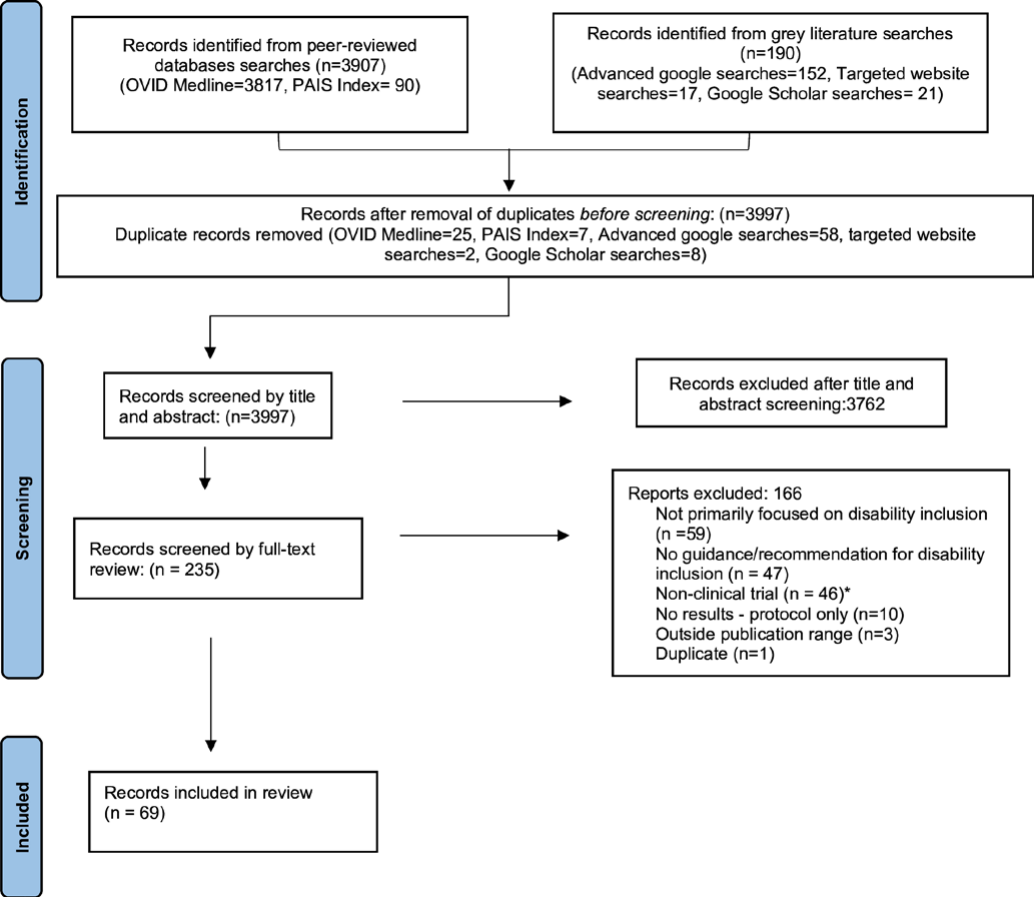
PRISMA study selection flow diagram showing the study selection process for the scoping review. PAIS, Public Affairs Information System; PRISMA, Preferred Reporting Items for Systematic Reviews and Meta-Analyses. *This project was a subset of a larger rapid scoping review with the aim of identifying guidance on disability inclusion in research (including but not limited to clinical trials). For this scoping review, the focus was solely on inclusion of people with disabilities in clinical trials and, as such, we pulled out any clinical trials related documents from the larger scoping review.

The documents from the OVID Medline and PAIS Index search strategies were imported into Covidence (Covidence systematic review software, Veritas Health Innovation, Melbourne, Australia) and duplicates were removed (n=3875) (figure 1). Reviewers were split into three pairs: GJ and FC (n=1291), GJ and VV (n=1291) and GJ and CC (n=1293). Any conflicts were resolved by a third reviewer. Peer-reviewed documents related to clinical trials were identified and abstracted from the larger scoping review (n=23).

For the grey literature search, reviewers were assigned unique Advanced Google searches across four domains: .edu, .ac, .gov and .org (online supplemental table 3). The targeted website and Google Scholar searches were conducted by GJ. Grey literature review and adjudication were conducted by the same pairs as for the OVID Medline and PAIS databases. The grey literature documents were imported to Covidence between 3 March 2025 and 15 April 2025, and duplicates were removed. The same reviewer pairs and adjudication strategies were used for the Google Scholar searches.

### Data analysis

The following characteristics were extracted into Covidence for the peer-reviewed and grey literature documents: country or region in which the policy/guidance originates, country or region in which the policy/guidance is aimed towards, scope of guidance (state/city, national, international, organisational/institutional, undefined), database, disability types, target audience, focus of recommendations (study planning/development, recruitment, data collection, data analysis, reporting and dissemination, institutional review board (IRB)/ethics, other) and conflict of interest disclosure. The adjudication process, as outlined above, was used. The extracted data were analysed, summarised (online supplemental tables 4–10) and used to develop recommendations and actions for disability inclusion in clinical trials.

### Patient and public involvement

People with disabilities are included as co-authors and were involved in the design, implementation, dissemination and reporting of this scoping review.

## Results

We identified 69 articles and documents (figure 1 and online supplemental table 11) that outlined guidance on the equitable inclusion of people with disabilities in clinical trials. These spanned peer-reviewed literature, grey literature, policy guidance, institutional reports and regulatory frameworks. These included geographic representation across North America, Australia, Africa and Europe and outlined recommendations across disability types, including people with IDD, mobility disabilities, sensory disabilities, mental health conditions and cognitive impairments.

Disability was defined in various ways in the included documents, reflecting both medical and social models. While the majority of documents did not explicitly define disability (n=50),^58 11 12 15^,^60^ 10 documents used more medicalised definitions that emphasise functional limitations, impairments and clinical diagnostic criteria (ie, Diagnostic and Statistical Manual of Mental Disorders and the International Classification of Diseases).^61^,^69^ The remaining nine documents used social models of disability (ie, WHO International Classification of Functioning, Disability and Health (ICF), the Washington Group on Disability Statistics and the United Nations Conventions on the Rights of Persons with Disability).^1070^,^77^

Our review of these documents yielded seven domains of recommendations (table 1): (1) inclusive study design, (2) accessible recruitment, (3) inclusive data collection, (4) equitable data analysis, (5) accessible dissemination, (6) ethical oversight and (7) supported consent. An eighth cross-cutting domain outlined the integration of people with disabilities as researchers and decision-makers throughout the clinical research process. These domains are outlined in table 1 and summarised below.

**Table 1:** Summary of domains and recommendations

Domain	Citing documents	Summary
Inclusive and universal study design	Kushalnagar *et al*^8^ 2023; Camanni *et al*^12^ 2023; McDonald *et al*^15^ 2024; McDonald *et al*^16^ 2022; Janevic *et al*^17^ 2022; Diemer *et al*^18^ 2022; Wang *et al*^63^ 2023; Shariq *et al*^10^ 2023; Mintz *et al*^19^ 2022; Frankena *et al*^20^ 2019; Witham *et al*^21^ 2020; MRCT Center 2023^71^; Shepherd (1) 2020^22^; Thompson *et al*^72^ 2020; Shepherd (2) 2020^47^; University of Washington n.d.^26^; DeCormier Plosky *et al*^5^ 2022; McDonald *et al*^65^ 2023; Cunningham *et al*^66^ 2025; Friesen *et al*^24^ 2023; WHO 2022^7^; Sadler 2023^25^; Bard 2021^74^; FDA(1) 2023^23^ Chen *et al*^67^ 2024; Northwestern University International Review Board^50^ n.d.; Andrews^27^ 2020; Sakuma *et al*^51^ 2024; ASH Clinical News^28^ 2021; National Council on Disability^2^ 2024; FDA (3)^29^; National Federation of the Blind^31^ n.d.; Routen *et al*^32^ 2022; Rutta^30^ 2024; Lamontagne *et al*^33^ 2021; Dubreuil *et al*^34^ 2024; Mishra *et al*^52^ 2025; Carneiro *et al*^35^ 2025; Leigh and Pillai^59^ 2025; Brathwaite 2024; Berg *et al*^53^ 2024; Kolbe^60^ 2024; Agaronnik *et al*^75^ 2025; Biggs *et al*^36^ 2024; Banas *et al*^77^ 2019; Schwartz and Unni^37^ 2021; Cockburn *et al*^54^ 2024; Ouellette *et al*^76^; Bradley^55^ 2021	Integrate universal design principles Planning and budgeting for accommodations from the inception of the project and grant writing Engage people with disabilities as co-designers Articulating clearly defined eligibility criteria to avoid automatic exclusion due to disability Use of equity frameworks and anti-ableist trainings during study design
Accessible and flexible recruitment	Kushalnagar *et al*,^8^ 2023; Meierer, 2022; McDonald *et al*,^15^ 2024; Piantedosi, 2023; MacNeil, 2024; Janevic *et al*,^17^ 2022; Diemer *et al*,^18^ 2022; Deckler *et al*,^40^ 2022; Wang *et al*,^63^ 2023; Shariq *et al*,^10^ 2023; Raskoff *et al*^64^ 2023; Deshpande *et al*^44^ 2020; Frankena *et al*^20^ 2019; Dakic^46^ 2020; MRCT^71^ 2023; Shepherd (1)^22^ 2020; Thompson *et al*,^72^ 2020; Shepherd (2)^47^ 2020; University of Washington^26^ n.d.; DeCormier Plosky *et al*^5^ 2020; McDonald *et al*^65^ 2023; Cunningham *et al*^66^ 2025; Friesen *et al*^24^ 2023; Sadler^25^ 2023; OCR^68^ 2023; FDA (2)^49^ 2023; Northwestern University Institutional Review Board Office^50^ n.d.; Andrews^27^ 2020; National Academy of Sciences^41^ 2022; Sakuma *et al*^51^ 2024; ASH Clinical News^28^ 2021; National Council on Disability^2^ 2024; University of Michigan^56^ 2023; FDA (3)^29^ 2023; National Federation of the Blind^31^ n.d.; Routen *et al*^32^ 2022; Rutta^30^ 2024; Lamontagne *et al*^33^ 2021; Dubreuil *et al*^34^ 2024; Carneiro *et al*^35^ 2025; Leigh and Pillai^59^ 2025; Brathwaite *et al*^11^ 2024; Kolbe^60^ 2024; Agaronnik *et al*^75^ 2025; Biggs *et al*^36^ 2024; Banas^76^ 2019; Ouellette^76^ 2019	Use multi-modal and culturally appropriate outreach methods Partner with trusted disability organisations Address logistical and communication barriers to equitable participation for people with disabilities Inclusion of people with disabilities as advisors, partners and collaborators during recruitment
Inclusive data collection	McDonald *et al*,^16^ 2022; Piantedosi, 2023; MacNeil 2024; Janevic *et al*,^17^ 2022; St. John *et al*,^62^ 2022; Wang *et al*,^63^ 2023; Frankena *et al*^20^ 2019; MRCT Center,^71^ 2023; Thompson *et al*,^72^ 2020; Shepherd (2)^47^ 2020; University of Washington^26^ n.d.; DeCormier Plosky *et al*^5^ 2022; Cunningham *et al*^66^ 2025; Friesen *et al*^24^ 2023; Sadler,^25^ 2023; WHO,^73^ 2022; Bard^74^ 2021; FDA(1)^23^ 2023; National Academy of Sciences^41^ 2022; National Council on Disability^2^ (2024); National Federation of the Blind^31^ n.d.; Routen *et al*^32^ 2022; Petersen^58^ 2023; Carneiro *et al*^35^ 2025; Biggs *et al*^36^ 2024; Banas *et al*^77^ 2019; Cockburn *et al*^54^ 2024; Ouellette^76^ 2019; Bardley^55^ 2021	Ensure data collection accounts for diverse access and communication needs Provide accessible and flexible data collection tools (ie, easy read formats, video, visual aids, plain language) Offer multiple modalities for data collection Inclusion of disability as a demographic element in data collection
Equitable data analysis	Janevic *et al*,^17^ 2022; Wang *et al*,^63^ 2023; Frankena *et al*,^20^ 2019; Witham *et al*,^21^ 2020; MRCT Center,^71^ 2023; University of Washington,^26^ n.d.; Biggs *et al*,^36^ 2024	Conduct disaggregated and intersectional analyses Include people with disabilities in the interpretation process Apply disability-inclusive analytic frameworks
Accessible reporting and dissemination	Janevic *et al*,^17^ 2022; Diemer *et al*,^18^ 2022; St. John *et al*,^62^ 2022; Frankena *et al*,^20^ 2019; Witham *et al*,^21^ 2020; MRCT Center,^71^ 2023; Thompson *et al*,^72^ 2020; University of Washington,^26^ n.d.; National Academy of Sciences^41^ 2022; National Council on Disability^2^ 2024; FDA (3),^29^ 2023; Rutta^30^ 2024; Carneiro *et al*^35^ 2025; Kolbe^60^ 2024; Biggs *et al*^36^ 2024; Banas *et al*^77^ 2019	Disseminate findings in plain language and accessible formats Ensure disability community-focused reporting strategies Engage people with disabilities in knowledge translation Protect participant confidentiality and ensure transparency of data reporting during dissemination of results Requiring minimum accessibility standards for reporting outputs
Ethics and institutional oversight	Meierer, 2022; McDonald *et al*,^16^ 2022; St. John *et al*,^62^ 2022; Shariq *et al*,^10^ 2023; Raskoff *et al*,^64^ 2023; Deshpande *et al*^44^ 2020; Frankena *et al*,^20^ 2019; Dakic,^46^ 2020; Witham *et al*,^21^ 2020; MRCT Center,^71^ 2023; Shepherd (1),^22^ 2020; Thompson *et al*,^72^ 2020; Shepherd (2)^47^ 2020; University of Washington^26^ n.d.; DeCormier Plosky *et al*^5^ 2022; McDonald 2023; Cunningham *et al*^66^ 2025; Friesen *et al*^24^ 2023; WHO,^7^ 2022; Wickremsinhe *et al*^48^ 2023; Bard^74^ 2021; UCSF^69^ 2023; Northwestern University Institutional Review Board Office^50^ n.d.; National Academy of Sciences^41^ 2022; ASH Clinical News^28^ 2021; National Council on Disability^2^ 2024; University of Michigan^56^ 2023; Silverman *et al*^57^ 2022; FDA (3),^29^ 2023; Rutta^30^ 2024; Dubreuil *et al*^34^ 2024; Carneiro *et al*,^35^ 2025; Leigh and Pillai,^59^ 2025; Banas *et al*^77^ 2019; Ouellette^76^ 2019	Include people with disabilities in IRBs and ethics boards Shifting from overly protectionist approaches to inclusive frameworks that balance protection with equitable access and participation Require clear justification for exclusion of people with disabilities from trials Align ethical review with rights-based frameworks such as the United Nations CRPD
Supported and accessible consent	Meierer, 2022; McDonald *et al*,^15^ 2024; McDonald *et al*^16^ 2022; Piantedosi, 2023; MacNeil 2024; Thurm *et al*^42^ 2022; Deckler *et al*^40^ 2022; St. John *et al*,^62^ 2022; Shariq *et al*,^10^ 2023; Raskoff *et al*,^64^ 2023; Russell *et al*,^43^ 2023; Deshpande *et al*,^44^ 2020; Dakic,^46^ 2020; MRCT Center,^71^ 2023; Shepherd (1)^22^ 2020; Shepherd (2)^47^ 2020; DeCormier Plosky *et al*^5^ 2022; Wickremsinhe *et al*^48^ 2023; FDA (1),^23^ 2023; UCSF^69^ 2023; FDA (2),^49^ 2023; ASH Clinical News^28^ 2021; National Council on Disability^2^ 2024; University of Michigan^56^ 2023; Silverman *et al*^57^ 2022; FDA (3),^29^ 2023; Carneiro *et al*,^35^ 2025; Leigh and Pillai^59^ 2025; Kolbe^60^ 2024; Agaronnik *et al*^75^ 2025; Biggs *et al*,^36^ 2024; Ouellette,^76^ 2019; Bradley^55^ 2021	Consent processes and materials that are accessible, including but not limited to flexible and supported decision-making Avoid presumption of inability to meet research competency standards Tailor procedures to individual needs and communication styles
Inclusion of people with disabilities as researchers	Camanni *et al*,^12^ 2023; Thurm *et al*,^42^ 2022; Heath and Levine,^70^ 2022; St. John *et al*,^62^ 2022; Wang *et al*,^63^ 2023; Mintz *et al*,^19^ 2022; Frankena *et al*,^20^ 2019; Nguyen *et al*,^45^ 2019; McDonald *et al*,^65^ 2023; Cunningham *et al*,^66^ 2025; WHO,^7^ 2022; Sadler,^25^ 2023; Northwestern University Institutional Review Board Office^50^ n.d.; Sakuma *et al*,^51^ 2024; Rutta^30^ 2024; Dubreil *et al*^34^ 2024; Berg *et al*^53^ 2024; Biggs *et al*^36^ 2024; Banas *et al*^77^ 2019; Cockburn *et al*^54^ 2024; Bradley,^55^ 2021	Engage people with disabilities as co-researchers and advisors Provide training and mentorship Promote meaningful participation of people with disabilities across all phases of research

CRPD, Convention on Rights of Persons with Disabilities; IRBs, institutional review boards.

### Inclusive and universal study design

Several articles (n=38) emphasised the need to embed universal design principles across all stages of clinical research. This includes proactive budgeting for accommodations (ie, use of interpreting agencies, extended time for assessments, longer study visits to allow for communication or mobility needs) and protocols that allow for flexibility and adaptability based on participants’ needs.^25 8 10^,^12 15^ Eligibility criteria should be defined using standardised and valid assessments, avoiding exclusion by default due to disability. Other sources (n=11) highlighted the role of anti-ableist and equity-focused frameworks in improving access and reducing systemic exclusion.^1550^,^55 65 66 73 75^ The engagement of people with disabilities as co-designers and collaborators in protocol development was consistently recommended.^17^,^1936 50 63 71 73^

### Accessible and flexible recruitment

Across the included studies, common recommendations were the availability of multiple recruitment modalities and culturally and linguistically accessible materials. Multiple documents (n=11) emphasised the importance of trust-building via engagement with advocacy organisations and networks.^1023 25^,^30 38^ Logistical challenges, such as with transportation, adequate time, childcare and literacy, were identified as barriers to increasing the participation of people with disabilities in trials.^5 17 18^ Recommendations also highlighted a need to involve people with disabilities as advisors, partners and collaborators during the recruitment process.

### Inclusive data collection practices

The documents reviewed identified best practices in data collection processes, including the need to accommodate varying communication, access needs and literacy levels. Suggested approaches include using alternative tools to support comprehension (ie, easy read formats, video, visual aids, plain language) and flexible scheduling or virtual formats to reduce participant burden.^16 20 22 35 38 41 71^ Stakeholder engagement in the design and piloting of data collection tools was advised to ensure inclusion and accessibility.^63^ Some sources (n=5) emphasised the need to avoid underreporting and ensure completeness of data across disability populations. This includes the need for standardised data collection tools that include the assessment of people with disabilities as a demographic.^2 16 32 54 55^

### Equitable data analysis

Guidance on data analysis encouraged inclusive and intersectional frameworks that allow for sample diversity. Five documents recommended pre-specifying subgroup analyses by disability status and, when possible, conduct analyses that provide insights into differential effects across subpopulations for which data are scarce.^17 21 26 36 71^ Involving people with disabilities and caregivers in data interpretation was highlighted as a strategy to improve the validity of clinical trials findings.

### Accessible reporting and dissemination

Dissemination plans should include community-accessible formats (ie, plain-language summaries, infographics, social media posts) and engage people with disabilities in the review and communication of results.^2 17 35 36 50 62 73 77^ Emphasis was placed on transparency (ie, publicly available dashboards, accessible data repositories), participant confidentiality, disaggregation of findings by disability subgroups and avoidance of deficit-oriented terms.^29 41 60 72^ Two documents also urged research funders and journals to enforce minimum accessibility standards for reporting outputs.^30 60^

### Ethics and institutional oversight

Inclusion of people with disabilities on IRBs and ethics committees was widely recommended. A key ethical consideration raised across studies was the need for IRBs to achieve a balance between protecting individuals with disabilities from harm and the ethical obligation to include them in research, as approaches solely emphasising protection can contribute to the routine exclusion of people with disabilities from research participation.^24 28 39 61 76^ In that regard, studies outlined the need to uphold the rights of people with disabilities to equitable access and participation in research. Additional recommendations were clear justification for exclusion to ensure non-discrimination and ethical inclusivity, flexible protocols to support informed participation, and training for IRB members on disability rights and supported decision-making.^2 5 29 34 35 44 46 50 57 61 64 66 71 73^ Four documents highlighted the need to align review process with rights-based frameworks and legal mandates (ie, the Americans with Disabilities Act or United Nations Convention on the Rights of People with Disabilities) to reinforce ethical accountability and support ethical mandates for accessibility.^10 48 64 74^

### Supported and accessible consent procedures

There was strong consensus on the need to improve consent processes to meet the needs of people with disabilities. This included providing consent materials in accessible formats (ie, plain language, visual aids), allowing extended time for decision-making and incorporating surrogate or supported decision-making only when appropriate.^10 15 16 38 61 62 71 75 76^ Researchers and ethical board members are advised to avoid assuming that people with disabilities cannot meet consent capacity standards; instead, individual assessments using tailored, appropriate methods should be used. We identified a set of studies (n=25) providing detailed guidance on ethical consent involving people with intellectual or cognitive disabilities, emphasising autonomy, presumption of competence and safeguards for assent and dissent.^25 15 16 22 23 28 29 35 38 39 42 43 47 49 55 57 59^,^62 64 69 71 75^

### People with disabilities as researchers and stakeholders

Ensure people with disabilities are included across the clinical trial process—not only as participants but as investigators, advisors and collaborators. This encompasses ensuring research teams not only include people with disabilities but also actively seek avenues to offer training and mentorship to people with disabilities, valuing the expertise and elevating the voices of people with disabilities.^12 19 20 30 42 45 51 54 62 70 73^ Co-production and power-sharing were further identified as critical components for disability-inclusive clinical trials.

To ensure that the evidence we have gathered is informative to facilitate disability-inclusive changes across different stages of clinical trials, we used the summary of guidance from each of the eight domains of recommendations (table 1) to develop action-oriented recommendations organised by the clinical trial stages in table 2.

**Table 2:** Recommended actions for creating disability inclusive clinical trials

Clinical trial stage	Action items
Trial planning and protocol development	Perform an assessment on the accessibility of trial sites, including evaluations of physical spaces, signage, restrooms and parking options. Following the assessment of trial site accessibility, allocate a dedicated budget for adequate training and accommodations to facilitate the inclusion of participants with disabilities, including sign language interpreters, extended visit times, accessible transportation and accessible materials. Involve people with disabilities as co-designers of trial protocols to inform design and accessibility. Integrate flexibility into study procedures by offering remote participation, adaptive scheduling and extended visit times to increase inclusion. Avoid disability-based exclusions unless scientifically justified.
Recruitment and participant engagement	Create recruitment materials in multiple accessible formats (plain language/easy read, sign language, video, large print, captioned videos). Ensure that all digital materials comply with established accessibility standards, such as the WCAG. Use multiple recruitment strategies, such as outreach through disability organisations, community events, accessible social media, SMS messaging and clinical settings. Provide an avenue to request accommodations during recruitment and offer transportation support, such as vouchers or accessible ride services. Provide flexible scheduling options, including evenings and weekends, and offer childcare support when feasible. Partner with and compensate disability advocacy groups to enhance cultural humility and accessibility of recruitment processes.
Consent and enrolment	Ensure all consent forms are in accessible formats, including plain language/easy read, screen-reader compatible and large print. Plan for extra time for participants to discuss and ask questions during consent. Provide communication supports, such as sign language interpreters, visual aids and augmentative communication devices. Avoid assuming people with disabilities cannot meet consent capacity standards. Use supported decision-making processes before allowing surrogate consent. Document procedures for obtaining assent and recognising dissent among participants with cognitive disabilities.
Data collection	Ask each participant about their communication and accessibility needs for data collection. Offer multiple data collection modalities (online, phone, video, in-person, text). Allow for flexible data collection sessions/structure (pauses, extended sessions, multiple short visits). Train data-collection staff in disability-inclusive communication techniques and cultural humility. Collect disability as a demographic variable using standardised measures. Pilot all data collection instruments and processes with people with disabilities.
Data analysis	Pre-specify analyses stratified by disability status. Conduct intersectional analyses considering multiple identities (eg, disability×race). Include people with disabilities or caregivers in data interpretation. Assess whether accommodations influenced outcome measures, such as subgroup analysis or sensitivity analyses.
Reporting and dissemination	Provide plain-language/easy-read summaries of results for participants and communities. Disseminate findings in accessible formats (infographics with alt text, captioned videos, accessible PDFs, Braille-ready files). Disaggregate results by disability status. Use rights-based, non-stigmatising language to describe people with disabilities. Document the accessibility features and accommodations used in the trial. Create accessible data repositories to ensure access by people with disabilities.
Ethics and institutional oversight	Include people with disabilities on IRBs and advisory committees. Require protocols to outline the scientific justification for any exclusion based on disability. Align protocols with disability rights frameworks and policies (eg, ADA/Section 504, CRPD). Require all trial proposals and protocols to include an accessibility plan. Ensure consent processes include autonomy-supportive approaches. Train IRB members in disability rights and supported decision-making.
Involvement of people with disabilities as researchers and stakeholders	Include people with disabilities as investigators, consultants or advisors and provide compensation for these roles. Ensure accessible work environments and communication systems. Incorporate participatory or co-production into trial methods. Budget time and resources for engagement and co-research activities.

CPRD, Convention on Rights of Persons with Disabilities; IRBs, institutional review boards; WCAG, Web Content Accessibility Guidelines.

## Discussion

This scoping review identifies common strategies for strengthening the inclusion of people with disabilities in clinical trials. Across 69 documents from around the world, we identified widespread recognition of the need to include people with disabilities in clinical trials and an emerging consensus on practical strategies across trial stages and processes. While some frameworks acknowledge the need for inclusive design, few offer enforceable requirements or detailed implementation strategies. Our review also identifies specific gaps, particularly in trial recruitment accommodations, ethical guidance for IRBs and accessible data dissemination, highlighting a need for further development of practices, policies and recommendations to improve the inclusion of people with disabilities in clinical trials.

Developing strategies to increase the inclusion of people with disabilities in clinical trials is an urgent public health issue. Excluding this population not only perpetuates health inequities, but also undermines the scientific validity and generalisability of clinical trials. Efficacy of trial interventions is not universal when 16% of the global population, or 1.3 billion people with disabilities, are excluded.^1^ For example, data indicate that people with Down syndrome have an increased risk of developing dementia and 90% to 100% of this population may develop Alzheimer’s disease after age 65.^78^,^80^ Yet, people with Down syndrome have been excluded from participation in multiple Alzheimer’s disease trials that led to U.S. Food and Drug Administration (FDA)-approved treatments.^81^ This exclusion makes it impossible to understand the safety and efficacy of Alzheimer’s drugs among a high-risk population.

While there may be valid scientific reasons to exclude certain individuals from clinical trials, these exclusions must be narrowly defined, scientifically justified and transparently reported to avoid discriminatory practices. Too often, exclusions of people with disabilities from clinical trials are based on broad categories or incorrectly attributed to a person’s impairment, such as intellectual disabilities, mental health conditions or physical impairments.^2 12 75 76^ Instead, potential participants should be evaluated individually, with careful consideration of whether accommodations can enable their participation. For example, publications by McDonald *et al*,^15^ Raskoff *et al*,^64^ Russell *et al*,^43^ the National Council on Disability,^2^ the University of California San Francisco^69^ and the University of Washington^26^ emphasise this need for clear justification of exclusion criteria to ensure people with disabilities are not unjustly excluded from trials.

Researchers may argue that accommodating diverse access needs—such as providing accessible transportation, communication aids or modified testing environments—adds complexity, cost and logistical burden, which could affect a trial’s feasibility. However, excluding participants solely due to their accessibility needs perpetuates systemic bias and often violates disability rights laws and protections in many countries.^10 48 64 74^ Instead, researchers must plan for accessibility from the outset and across the clinical trial process—through universal design, flexible protocols and dedicated resources or line-item budgets. MacNeil *et al*,^39^ MRCT Center,^71^ University of Washington,^26^ Biggs *et al*^36^ and Banas *et al*^77^ all identify and provide recommendations for addressing accessibility needs during the study planning and implementation of clinical research. It should be noted that this review focused on the common clinical trial barriers that people with disabilities face. However, people with different types of disabilities often face unique barriers that may necessitate specific interventions and approaches.

The WHA resolution (WHA 75.8), Strengthening Clinical Trials to Provide High-Quality Evidence on Health Interventions and to Improve Research Quality and Coordination, affirms that the inclusion of under-represented populations is essential for ensuring equity as a central principle of clinical trials.^7^ However, despite this commitment, there remains a disconnect between principle and practice. There have been few, if any, concrete steps toward ensuring the resolution’s objectives for people with disabilities. Structural barriers within research systems continue to exclude this population, and it is clear that deliberate, systemic action is now needed.

This review outlines a set of steps and existing practical measures that can help translate the WHA 75.8 resolution into practice by addressing the barriers to including people with disabilities in trials. Such progress is feasible, as demonstrated by the study conducted by Deckler *et al*,^40^ which presents a recruitment model for engaging individuals with schizophrenia—a group often excluded from clinical trials. By implementing strategies such as hiring a dedicated recruiter, conducting targeted chart reviews at external affiliated clinics and developing an umbrella protocol to streamline consent and screening, this clinic was able to increase participant enrolment by approximately 40%.^40^ This example illustrates that with thoughtful design, institutional support and proactive engagement strategies, the inclusion of people with disabilities in clinical research is both achievable and effective.

### Strengths and limitations

This review’s strengths include its inclusion of peer-reviewed and grey literature, adherence to the Arksey and O’Malley scoping review framework and reporting according to PRISMA-ScR. Limitations of this review include restriction to English-language documents, exclusion of unpublished or non-written resources and rapid review timeframes. Due to the rapid review timeframes, this review was limited to two peer-reviewed databases. However, we believe that the specific nature of our research aim and the inclusion of a broad biomedical database (OVID Medline), a subject specific database (PAIS Index) and multiple grey literature searches (custom advanced Google searches, targeted website searches and Google Scholar search) provided a sufficient overview of documents related to disability inclusion in clinical trials.

## Conclusion

The inclusion of people with disabilities in clinical trials remains a deprioritised area of research. While there is a growing number of policies and guidance on how to improve the inclusion of people with disabilities in clinical trials, there is still a need to synthesise this knowledge and create a roadmap for disability inclusion across all steps in the clinical trial process. This guidance must be grounded in principles of equity and human rights and will require the support of national governments, research institutions and ethics boards to enforce disability-inclusive clinical trial practices.

## Supplementary material

10.1136/bmjopen-2025-108550 online supplemental file 1

## Data Availability

All data relevant to the study are included in the article or uploaded as supplementary information.
