# Supplementary material for: Equitable inclusion of people with disabilities in clinical trials: a scoping review
Source: BMJ Open. 2026 Feb 4;16(2):e108550. doi: 10.1136/bmjopen-2025-108550 (PMC12878309; doi:10.1136/bmjopen-2025-108550)
Supplement: online supplemental file 1 [file bmjopen-16-2-s001.docx]

# Supplementary Material

**Supplemental Table 1: OVID MEDLINE Search Strategy**

| **Step** | **Search terms** | **Number of results** |
| --- | --- | --- |
| 1 | (disab* or (function* adj1 limitation*)).ti,ab,kf,kw or (exp disabled persons/) | 359480 |
| 2 | (Consensus/ or exp Consensus Development Conference/ or Consensus Development Conferences as Topic/ or exp Guidelines as Topic/ or exp Guideline/ or exp Policy/ or exp Models, Theoretical/ or exp Checklist/) or (“Consensus Development” or Policy or policies or guideline* or recommend* or “best practice*” or mandat* or checklist* or tool* or toolkit* or checklist* or parameter* or guidance).ti,ab,kf,kw. | 6012260 |
| 3 | ((exp Diversity, Equity, Inclusion/) and (research/ or exp Community-Based Participatory Research/ or exp Stakeholder Participation/)) or ((inclusion* or inclusiv* or accommodat* or particip* or stakeholder*) adj5 research*).ti,ab,kf,kw. | 54166 |
| 4 | (Exp Government/ or exp International Agencies/) or (government* or “Advocacy Group*” or agency or agencies or NGO or CDC or NIH or UN or “United Nations” or “World Health Organization” or “Centers for Disease Control” or Institute* or (National adj2 Center*)).ti,ab,kf,kw. | 868115 |
| 5 | 1 and 2 and (3 or 4) | 9956 |
| 6 | limit 5 to (English language and yr="2019-current") | 3817 |

*Since the development of this search strategy, the Mesh term “exp disabled persons/” was updated to “exp Persons with Disabilities/”

**Supplemental Table 2: PAIS Index (ProQuest) Search Strategy**

| **Step** | **Search terms** | **Number of results** |
| --- | --- | --- |
| 1 | MAINSUBJECT.EXACT.EXPLODE("People with disabilities") OR noft(disab* OR (function* NEAR/1 limitation*)) | 15,547 |
| 2 | ((MAINSUBJECT.EXACT.EXPLODE("Diversity training") OR MAINSUBJECT.EXACT.EXPLODE("Equity")) AND (MAINSUBJECT.EXACT.EXPLODE("Organizational research") OR MAINSUBJECT.EXACT.EXPLODE("Qualitative research") OR MAINSUBJECT.EXACT.EXPLODE("Research ethics") OR MAINSUBJECT.EXACT.EXPLODE("Health research") OR MAINSUBJECT.EXACT.EXPLODE("Research") OR MAINSUBJECT.EXACT.EXPLODE("Community research"))) OR noft((inclusion* OR inclusiv* OR accommodat* OR particip* OR stakeholder*) NEAR/5 research*) | 6024 |
| 3 | 1 and 2 | 208 |
| 4 | limit 3 to (English language and yr="2019-01-01 – 2024-10-18") | 90 |

*Since the development of this search strategy, the term MAINSUBJECT.EXACT.EXPLODE("People with disabilities") was updated to MAINSUBJECT.EXACT.EXPLODE("Disabled people")

**Supplemental Table 3: Advanced Google Search Strategies**

| **Search Strategy 1** | **Search terms** |
| --- | --- |
| All these words: | persons with disability |
| This exact word of phrase: | clinical research |
| Any of these words | guidance recommendation including excluding |
| None of these words:* |  |
| Numbers Ranging from:* |  |
| Language | English |
| Region | any region |
| Last update: | anytime |
| Site or domain:** | .edu/.ac/.gov/.org |
| Terms appearing: | anywhere in the page |
| File type: | any format |
| Usage rights: | not filtered by license |
| Search:*** | persons with disability guidance OR recommendation OR including OR excluding "clinical research" site:.edu |
| **Search Strategy 2** | |
| All these words: | disability research |
| This exact word of phrase: | clinical research |
| Any of these words | accessibility inclusion recommendation guidelines |
| None of these words: |  |
| Numbers Ranging from: |  |
| Language | English |
| Region | any region |
| Last update: | anytime |
| Site or domain: | .edu/.ac/.gov/.org |
| Terms appearing: | anywhere in the page |
| File type: | any format |
| Usage rights: | not filtered by license |
| Search: | disability research accessibility OR inclusion OR recommendation OR guidelines "clinical research" site:.edu |
| **Search Strategy 3** | |
| All these words: | disability |
| This exact word of phrase: | clinical trials |
| Any of these words | policy ethics advisory committee |
| None of these words: |  |
| Numbers Ranging from: |  |
| Language | English |
| Region | any region |
| Last update: | anytime |
| Site or domain: | .edu/.ac/.gov/.org |
| Terms appearing: | anywhere in the page |
| File type: | any format |
| Usage rights: | not filtered by license |
| Search: | disability policy OR ethics OR advisory OR committee "clinical trials" site:.edu |

*these were intentionally left blank to not limit the search

**each site or domain was searched separately (i.e. for Search Strategy 1, four advanced google searches were completed (one for each domain))

***only the full search of the .edu domain is shown in the table

**Supplemental Table 4: Study Planning/Development Recommendations**

| Number | First Author | Year | Recommendation (Summary) |
| --- | --- | --- | --- |
|  | Kushalnagar | 2023 | More inclusive study design/planning during grant development/protocol design. Budgeting for accommodations, utilization of interpreting agencies, effective training, and building in flexibility into the protocol. Educating medical professionals about deaf health disparities and providing accessible tools to facilitate the referral of deaf patients to clinical trials. |
|  | Camanni | 2023 | Universal research design, accommodations/modification, and inclusion of people with disabilities (pwd) in research development. Clearly specifying and justifying inclusion and exclusion criteria to avoid vague/broad criteria that disproportionately exclude PWDs. Minimizing the use of exclusion criteria based solely on disabilities or conditions that could be accommodated through minor modifications in study methodology. |
|  | McDonald | 2024 | Utilizing anti-ableist research frameworks, community-engaged research design, and skill development for researchers to appropriately include pwd. Avoiding categorical exclusions based on intellectual disability and assessing individual capacity instead. |
|  | McDonald | 2022 | Developing adequate eligibility criteria that are precisely articulated and are grounded in standardized and valid assessments, as well as designing research protocols with inclusive accommodations and appropriate eligibility criteria in mind. Avoid default exclusion based on disability. |
|  | Janevic | 2022 | Including pwd in the design and decision-making process. Recommendations for community engagement in developing and designing research protocols, utilizing universal design, and forming partnerships. |
|  | Diemer | 2022 | Incorporating pwd (autistic children and people with intellectual disabilities) into the planning/development of the research question, partnership with the community, and incorporation of pwd into the research team. Journal editors and grant reviewers to require justification for any exclusion during evaluation/study design. Developing study protocols with pwd in mind (i.e. building autism diagnostic evaluations as a feature of the protocol during study development). |
|  | Wang | 2023 | Including children with disabilities and their families as co-researchers in designing and engaging disability research. This includes involving pwd in establishing engagement protocols/goals. |
|  | Shariq | 2023 | Increasing collaboration and co-design with pwd and partnership with the community in protocol/study design process. This includes utilizing co-design and community consultation in the study design research phase to develop accessible research protocols. |
|  | Mintz | 2022 | Inclusion of pwd as consultants, advisors, and team members to be consulted during study design and research implementation. This includes focusing on outreach to advocacy organizations and building accommodations into the research design, recruitment, intake, and follow-up assessment. |
|  | Frankena | 2019 | Accessible information regarding study design including modifications, clear communication, team responsibilities, financial compensation, and an accessible abstract included in study design/preparation. Plan for practical aspects such as transportation, accessible materials, and financial compensation in advance. |
|  | Witham | 2020 | Inclusive and accessible research design with flexible study protocols, accessible materials, multiple modalities, and adequate time/funding. |
|  | MRCT Center | 2023 | Implementing universal design in all aspects of clinical research from development, recruitment, data collection, data analysis, reporting and dissemination, and IRB/ethical concerns. This includes using clear communication, conducting site assessments for physical accessibility, providing adequate accommodations, training, extra time, and budgeting/funding for accessible research. Involving pwd in all aspects of the research process (including advocacy and dissemination). |
|  | Shepherd (1) | 2020 | Developing accessible clinical trial designs pertaining to informed consent for adults lacking capacity to consent, especially when considering surrogate consent, deferred or waiver of consent and emergency research consent. This includes prospectively appointing surrogates for research decisions, creating advance research directives (ARDs), and greater transparency in the research process. |
|  | Thompson | 2020 | Including children with disabilities into the study design/planning process as well as providing adequate accommodation for making the research accessible. This includes involving organizations representing or supporting people with disabilities for support and guidance on developing study design, data collection, data analysis, and research communication. Recommendations for developing “disability-inclusive child protection/safeguarding protocol” which protects the rights of children with disabilities, which includes researcher training in safeguarding policies and provisions for a formal complaints procedure |
|  | Shepherd (2) | 2020 | Recommendation for “patient-centered” approach in trials such as focusing on flexibility, and increased education and training for researchers in designing and conducting clinical trials with adults lacking capacity to consent. |
|  | University of Washington | n.d. | Designing studies with accessibility in mind including building in adequate time for consent and other research processes, accessible location and flexibility in hours and location for study visits), accessible materials, etc. |
|  | DeCormierPlosky | 2022 | Incorporating accessibility/accommodation needs in the study planning phase. This includes clear justification for any exclusions, using objective criteria for exclusion on an individual basis as opposed to broad exclusions, accessible materials and procedures, adequate modifications and clear terminology and language built into the study design of the study. |
|  | McDonald | 2023 | Utilizing universal design in all aspects of the study. This includes building in accessibility and accommodations into the study, and developing  anti-ableist research protocols that engage the disability community |
|  | Cunningham | 2025 | Recommendation for Universal design and use of Accessibility by Design Toolkit when planning and developing the research study. |
|  | Friesen | 2023 | Adapting study protocols with adequate accessibility and flexibility for inclusion of pwd in research. This includes partnering with community partners, justifying exclusion criteria, and providing accessible study materials. |
|  | WHO | 2022 | Including pwd in research team and in all phases of the research design process. Facilitating engagement through integrating universal design in all phases of the research or by providing reasonable accommodation throughout. Provide researchers and reviewers with Convention on the Rights of Persons with Disabilities (CRPD) training., which sets out the right of persons with disabilities to participate in issues that affect them, including health research. |
|  | Sadler | 2023 | Recommendations for universal design, accommodations, and modifications to be built in during the research development phase. This includes accessibility strategies such as screen-reader compatible materials, large, contrasting print, and plain language. |
|  | Bard | 2021 | Legal requirements for provision of accommodations and accessible research study designs. This includes building in adequate accommodations for the informed consent protocol/design and partnering with community stakeholders to involve pwd in the research design and implementation process. |
|  | FDA (1) | 2023 | Including pwd in the planning and development of clinical studies. This includes requiring scientific justification for any exclusion criteria for this population and partnering with community advocates to build trust and review research protocol. |
|  | Chen | 2024 | Recommendation for the precise definition of eligibility criteria, explicit justification for any exclusion of people with disabilities in research, and the inclusion of pwd in study design with considerations for accommodations, and accessibility., etc. |
|  | Northwestern University Institutional Review Board Office | n.d. | Including pwd in the research team and in the planning and conducting of research. Ensuring accessibility through accommodation and assistive technology, providing adequate exclusion justification, and utilizing Accessibility by Design Toolkit in planning and study development. |
|  | Andrews | 2020 | Applying universal design principles, partnering with pwd in all stages of the clinical trials process including study design and accessibility and justification for exclusion criteria. |
|  | National Academy of Sciences | 2022 | Building trust with the disability community and including pwd in the design of study with a focus on recruitment strategies for pwd, and considering accessibility barriers in the research protocol. |
|  | Sakuma | 2024 | Utilization of tools like the Impaired Capacity to Consent Framework in designing research study to improve accessibility and implementation. |
|  | ASH Clinical News | 2021 | Flexibility and accommodations built into the informed consent process with consultation with pwd. This includes building in accessible materials such as providing visuals for documents, incorporating feedback from pwd, and providing adequate time and resources for the research process. |
|  | National Council on Disability | 2024 | Accessible study design during research protocol development including lists of accommodations, explicit eligibility criteria with justification for any exclusions, clear language, etc. |
|  | FDA (3) | 2023 | Involving pwd in the study design phase of clinical trials to build trust, partnership and build in proper accommodations and accessibility tools into the research protocol. |
|  | National Federation of the Blind | n.d. | Including accessibility in the research process by building in accommodations in the study design and including pwd as researchers. |
|  | Routen | 2022 | Building cultural competency among research team and including pwd in study design and adapting their designs for more inclusive resources, budgets, and resources. |
|  | Rutta | 2024 | Partnering with pwd for developing inclusive research study design which addresses increasing diversity in clinical trials. This includes developing and implementing public education about clinical trials, public awareness campaigns, building community-based partnerships, and utilizing innovative trial designs. |
|  | Lamontagne | 2021 | Appropriate accommodations, training, and co-design for involving perspective of people with traumatic brain injury in strategies and study protocols. |
|  | Dubreuil | 2024 | Develop accessible and multi-strategic study design for the inclusion of pwd in clinical research. This includes “designing research materials in a culturally competent manner”, “engage community members early in the research process to address concerns and thereby promote trust and willingness to participate”, providing adequate compensation and building flexibility into the research protocol. |
|  | Mishra | 2025 | Identifies various research design frameworks that consider equity. For example, “using the Equity by Design framework to support design of equity focused and inclusive trials that maximizes opportunity for participation of historically marginalized groups.” Other frameworks include Campbell & Cochrane Equity EQUITY guidelines and the Include Ethnicity Framework. |
|  | Carneiro | 2025 | Use of accessible “procedures, methods, language, tools, and spaces in the process of recruitment, conducting research, and research dissemination”. This includes building community partnerships and including trained and educated disability support staff. |
|  | Leigh | 2025 | Including pwd in the planning and development stages of research. This includes partnering with stakeholders, community organizations, and creating of regulatory guidelines including the need for “global strategies...to be developed to harmonize clinical studies with vulnerable populations.” |
|  | Brathwaite | 2024 | More diverse study protocol design (including multi-modal locations), less restrictive inclusion/exclusion criteria, and built in reimbursement/inclusive travel plans. |
|  | Berg | 2024 | Use of an anti-ableist framework. Receive and implement feedback and provide accessible materials. |
|  | Kolbe | 2024 | Some federal agencies consider incentives to sponsors and develop diversity action plans. Departments should provide equitable compensation to research participants. HHS should develop strategies to coordinate inclusion across Departments and build trust with pwd through improving transparency and accountability. |
|  | Agaronnik | 2025 | Build in creative accommodations and utilization of Accessibility by Design Toolkit, and clear justification for exclusion criteria. |
|  | Biggs | 2024 | Hire staff from underserved groups, train staff to have disability cultural competency, communicate with participants throughout the study, ensure exclusion criteria does not unnecessarily remove underserved groups from study, plan for diverse populations at the very start, and compensate participants fully and quickly. |
|  | Banas | 2019 | Plan to address participant mistrust in research |
|  | Schwartz | 2021 | Plan for accommodations from the start, such as providing interpreters, text-to-speech technology, mobility accessibility. Provide clear justification for exclusion criteria. |
|  | Cockburn | 2024 | Consider various frameworks of disability and use multiple when possible. This includes considering the context, terminology, and accessibility of methods. Select frameworks that will allow you to make the comparisons you want.  Train staff to understand these frameworks. Consider the costs of the study upfront. |
|  | Ouellette | 2019 | Implementing universal study design and provide accommodations to participants. Limit exclusion criteria to what is scientifically and ethically responsible., |
|  | Bradley | 2021 | Participatory Action Research strategies involve PWD in the development of science that affects them.  Promote the Right to Science in the Convention on the Rights of People with Disabilities. |

**Supplemental Table 5: Recruitment Recommendations**

| Number | First Author | Year | Recommendation (Summary) |
| --- | --- | --- | --- |
| 1. | Kushalnagar | 2023 | Accessible study recruitment materials, protocols, trainings, and community outreach. Providing “accessible tools to effectively refer deaf patients to clinical trials”, and promoting outreach efforts to encourage community engagement and research recruitment. |
| 2. | Meierer | 2022 | Self-advocacy and community input of pwd in research participation to allow for more relevant findings and greater participation for adults with ID in research. Providing adequate time for researchers “to include individuals belonging to marginalized communities”. Ensuring recruitment materials are designed to be understandable and accessible to individuals with intellectual disabilities, using clear and appropriate language. |
| 3. | McDonald | 2024 | More accessible recruitment materials including providing adequate accommodation and building in flexibility into the recruitment protocols. Ensuring that recruitment settings are familiar and comfortable to reduce perceived coercion. |
| 4. | Piantedosi | 2023 | Recruitment is built upon trust, partnership, and relationship building. This includes involving pwd and disability organizations in the recruitment process, incorporating participant feedback, and modifying materials to be more accessible. Considering strategies such as allowing participation during regular meetings of pre-existing groups, to reduce burden and to foster familiar environments for recruitment. |
| 5. | MacNeil | 2024 | Inclusive and accessible recruitment methods including using multiple recruitment modalities, flexible recruitment approach, stipends, accessible language and materials, and partnerships. Importance of collaborating with a)parents as experts on their child’s needs and behaviors to support personalized engagement, b)”gatekeepers” (e.g., school staff such as school principal, administrators, etc.) to build trust and facilitate recruitment by clarifying their role while addressing concerns about bias or pressure. |
| 6. | Janevic | 2022 | Involve advisory boards in shaping recruitment strategies and materials to ensure relevance and accessibility. Address barriers like transportation, child/elder care, work hours, and legal concerns (e.g., allow pseudonyms and non-SSN-based compensation). Offer culturally appropriate materials, inclusive imagery, and multiple modes of outreach (e.g., town halls, social media, mHealth). Engage family and community gatekeepers respectfully, and allow them to participate in information sessions. |
| 7. | Diemer | 2022 | Mitigate recruitment barriers by providing support such as transportation, childcare, etc. Increase outreach to populations through inclusive practices and materials that reflect intersectional identities. |
| 8. | Deckler | 2022 | Relationship building, staff members dedicated to recruitment, and community partnership/outreach to build trust. Prioritizing effective communication with providers and preparing site-specific materials (e.g., study summaries, flyers) |
| 9. | Wang | 2023 | Utilizing multiple modalities for recruitment and including stakeholders in meaningful roles during recruitment process. This includes utilizing social media platforms to build research partnerships and incorporating feedback from stakeholders during the recruitment process. Leverage existing engagement-focused organizations or consultation groups to recruit youth and families. |
| 10. | Shariq | 2023 | Involving pwd as researchers in the recruitment process. Providing adequate time and flexibility within the recruitment protocol, providing accessible materials, partnering with pwd/community organizations, and utilizing a person-centered approach to consent.  Collaborating with trusted local organizations and individuals familiar with the target disability population to enhance trust and recruitment pathways. |
| 11. | Raskoff | 2023 | Guidance for researchers to properly justify participation and recruitment selection with clear communication and consent process for fair participant selection. |
| 12. | Deshpande | 2020 | Guidance in how to ethically recruit participants if preferences of “mentally ill patients” are not aligned with their chosen surrogates. Recruitment of institutionalized individuals must ensure that participation is relevant to the study and that refusal is genuinely possible.  Guidance on payment/incentives is also given. |
| 13. | Frankena | 2019 | Continually optimizing recruitment strategies through feedback, providing training for team members, providing adequate time and funding/compensation for recruitment. |
| 14. | Dakic | 2020 | Engaging stakeholders, partnering with stakeholders and community organizations to consult in risk assessment for participant/recruitment into research. |
| 15. | MRCT Center | 2023 | Implementing universal design in all aspects of clinical research from development, recruitment, data collection, data analysis, reporting and dissemination, and IRB/ethical concerns. This includes using clear communication, providing extra time during the recruitment process, ensuring adequate funding, accommodations, and modifications for recruitment materials, and collaborating with pwd in all aspects of the research process, as well as  including pwd in the research team,). |
| 16. | Shepherd (1) | 2020 | Conducting ethical and inclusive enrollment/recruitment of participants especially in emergency situations. This includes being aware of consent-based recruitment biases, timely notification to the participant/the participant’s representative and involvement of the community in consultation in how and in what manner the research is conducted. |
| 17. | Thompson | 2020 | Partnering with local communities in order to recruit child participants. This includes engaging local children and disability organizations, using census/other sources of data on children with disabilities, utilizing various communication technology (including social media) for recruitment, and engaging with families to overcome “issues of stigma”. Recruitment strategies should also be alert to the potential for over-enrolment of children with certain characteristics who have participated in multiple studies. |
| 18. | Shepherd (2) | 2020 | Providing necessary time, funding, and accessible materials/training for pwd in clinical trials. |
| 19. | University of Washington | n.d. | Inclusive consent process in recruitment/enrollment decision and including flexible recruitment options. This includes involving trusted family, friends, or Legal Authorized Representative (LAR) of the participant during the recruitment process, considering alternatives to in-person recruitment such as electronic communications, and expanding recruitment to span rural areas |
| 20 | DeCormierPlosky | 2022 | Accessibility in the recruitment process including providing accommodation with ASL, clear communication, accessible study materials, accessible transportation to the study site etc. |
| 21. | McDonald | 2023 | Accessible recruitment materials and community perspectives on recruitment/consent protocols. |
| 22 | Cunningham | 2025 | Accessible recruitment and retention strategies utilizing Accessibility by Design Toolkit and STEP UP Guidance. |
| 23 | Friesen | 2023 | Inclusive recruitment plans and procedures with built-in flexibility and accessible materials. This includes clear justification for exclusion criteria and providing adequate accommodations (i.e. translations). |
| 24 | Sadler | 2023 | Partnering with community networks in order to increase participation, build trust, and increase accessibility and clarity in research. |
| 25. | OCR | 2023 | Justification for study exclusion from recruitment/participation in clinical trials. This includes providing alternative forms of communication, modifications, and tools for accessibility. |
| 26. | FDA (2) | 2023 | Recommendation for recruiting and enrolling pwd in clinical research. This includes provision of accessible materials, multiple modalities, accommodation, etc. |
| 27 | Northwestern University Institutional Review Board Office | n.d. | Providing accessible materials for recruitment. This includes involving pwd in the development and implementation of the recruitment protocol, providing accessible communication materials, and reviewing research protocol to encourage disability inclusion. |
| 28. | Andrews | 2020 | Utilizing universal design to create diversity in recruitment and consent.  This includes providing accessible materials, adequate diversity in recruitment, and engagement with the disability community to build trust and increase engagement. |
| 29. | National Academy of Sciences | 2022 | Building partnership, accessibility, and flexibility for recruitment and data collection. This includes building rapports with participants, providing adequate accessibility for research materials, involvement of community representatives/stakeholders, and adequate time/funding, |
| 30 | Sakuma | 2024 | Inclusive and accessible recruitment methods including multiple modalities, inclusion of stakeholders in developing recruitment protocol, and use of disability assessment tools. |
| 31 | ASH Clinical News | 2021 | Building trust and partnership with pwd and their families for more inclusive recruitment process. This includes building in adequate time, resources, and tools for inclusive participation. |
| 32 | National Council on Disability | 2024 | Accessible recruitment materials and multi-modality recruitment methods. This includes accessible recruitment websites, utilization of plain language, adequate funding, listing available accommodations and closed captioning. |
| 33 | University of Michigan | 2023 | Providing proper accommodation and accessibility modification for the participation, enrollment, and recruitment of pwd. This includes providing interpreters, accessible materials (alternative languages, alternative formats, etc.), and providing clear exclusion justification for the recruitment process. |
| 34. | FDA (3) | 2023 | Accessible recruitment materials, adequate time and funding, and proper accommodations when developing and implementing proper recruitment protocol. This includes involving pwd on the research team in the recruitment process in order to build trust and relationships with the community. |
| 35. | National Federation of the Blind | n.d. | Providing accommodation and proper funding for accessible recruitment for pwd. |
| 36. | Routen | 2022 | Utilizing toolkits and partnering with pwd to develop and implement inclusive and engaging recruitment protocols. |
| 37. | Rutta | 2024 | Inclusive enrollment with Diversity Action Plans and recruitment including building partnership, trust, and building adequate accessibility tools and strategies for increased participation for pwd in clinical research. “This could include the use of plain language, visual aids, and interactive tools that help convey key information in a clear and engaging way.” |
| 38. | Lamontagne | 2021 | Accessible training and strategies for participation in clinical trials. |
| 39. | Dubreuil | 2024 | More accessible recruitment materials including incentives, remunerations, requirements, and clear language open to those with limited "general and health literacy" |
| 40. | Carneiro | 2025 | Use of accessible “procedures, methods, language, tools, and spaces. This includes providing appropriate training to research staff and collaborating with community organizations in the recruitment process. |
| 41. | Leigh | 2025 | More inclusive recruitment strategies including “in-person recruitment, flexibility, and rapport building by researchers, with financial incentives in certain cohorts shown to improve recruitment rates.” |
| 42. | Brathwaite | 2024 | Inclusive recruitment methods including utilizing decentralized clinical trials, “implementing technology into clinical trial design ...allow[ing] existing rural hospitals to expand their patient reach”, incentivizing physicians to conduct clinical research, reimbursement/economic strategies for inclusive recruitment, and increased public education/outreach. |
| 43. | Kolbe | 2024 | Partner with community organizations to design research studies with “with the intention to recruit and retain diverse research participants”. This includes incorporating feedback/real-time adjustment to recruitment methods. |
| 44. | Agaronnik | 2025 | Researchers should use specific language when documenting reasons for excluding individuals so as to avoid default exclusion all people with disabilities |
| 45 | Biggs | 2024 | Build community partnerships, recruit from broad areas and in places familiar to your population, seek out rural populations, make recruitment sites accessible, adequate time and funding, multiple recruitment setting, using plain language, and proper training, and flexible recruitment protocol. |
| 46 | Banas | 2019 | Use multiple accessible recruitment materials and strategies. |
| 47 | Ouellette | 2019 | Accessible recruitment materials and accommodations (i.e. interpreters, assistive technology, and clear communication support recruitment of PWD. |

**Supplemental Table 6: Data Collection Recommendations**

| Number | First Author | Year | Recommendation (Summary) |
| --- | --- | --- | --- |
| 1. | McDonald | 2022 | Appropriate accommodations for data collection. This includes accessible materials (i.e. text-to –voice/voice-to-text technology), adequate time and flexibility built into the data collection procedure. Consider and plan for inclusive practices that reduce attrition and increase data completeness among diverse participants. |
| 2. | Piantedosi | 2023 | Easy read formats, plain language, multiple communication formats, etc. Accounting for participants’ preferences, including reading items aloud, allowing elaborations, and providing clarification on Delphi questions. |
| 3. | MacNeil | 2024 | Using multiple data collection methods and empowering participants. This includes accessible materials and alternative data collection methods (i.e. pictures, observations).  Addressing gaps in ethical guidelines that do not adequately cover populations with intersecting vulnerabilities (e.g., cognitive disability + childhood). |
| 4. | Janevic | 2022 | Adequate compensation (travel funds, transportation), accommodations, and accessible materials (text-to-speech, adequate lighting, clear communication, etc.) for inclusive data collection. Plan for diverse language needs, using translated and validated measures when possible and bilingual staff when necessary. |
| 5. | St. John | 2022 | Accessible data collection materials and protocols (i.e. accessible research locations, accommodations throughout research procedures, multiple data collection formats, etc.) |
| 6. | Wang | 2023 | Clear communication, partnership, and inclusion of pwd into data synthesis/collection. Engage stakeholders in interpreting findings by integrating their experiential knowledge to enhance relevance and nuance of conclusions. Use collaborative discussions between researchers and stakeholders to refine emerging themes and prioritize relevant content for reporting. |
| 7. | Frankena | 2019 | Providing training, utilizing alternative means of data gathering (i.e. video, visual data), inclusion of pwd in identifying accessible data collection methods. |
| 8. | MRCT Center | 2023 | Implementing universal design in all aspects of clinical research from development, recruitment, data collection, data analysis, reporting and dissemination, and IRB/ethical concerns. This includes protecting confidentiality during data collection, developing templates with standardized disability questions, providing flexible strategies that enable all participants to adhere to the expectations of the study (for example- accessibility, accommodations, extra time, transportation, reimbursement).  Collaborating with pwd in all aspects of the research process. |
| 9. | Thompson | 2020 | Involving children with disabilities in the planning of the data collection methodology as well as making all data collection material accessible, using approaches to aid the child’s understanding such as the use of drawings/symbols, photography and computer-aided approaches with access adaptations, and allowing sufficient time for data collection activities |
| 10. | Shepherd (2) | 2020 | Improving accessibility and accommodation in the data collection process through the use of decision and visual aids, revisiting consent throughout the data collection process, and utilizing flexible approach to data collection. |
| 11. | University of Washington | n.d. | Accessible materials for data collection including multiple modalities, alternative text, and images in data collection process. Also, combining study visits with existing clinic visits, limiting in-person visits and considering alternatives such as via tele-health visits. |
| 12 | DeCormierPlosky | 2022 | Providing accommodations in the data collection process including alternative and accessible test formats, virtual visits, adequate time, ASL, clear communication, and accessible transportation to the study site |
| 13 | Cunningham | 2025 | Collecting disability data in the data collection process. This includes discussion on a universal definition of disability in order to determine if disability is collected as demographic data or health status. |
| 14. | Friesen | 2023 | Increasing diversity and inclusion in participation and data collection process including providing accessible materials, continual assessment and review, and assessment if changes should be made mid-study to ensure diversity. |
| 15. | WHO | 2022 | Including pwd in all aspects of the research process including the research design, protocol development, participation, ethical committees, etc. This includes standardized data collection methods, selection of appropriate data collection tools, and integrating disability in data collection. |
| 16. | Sadler | 2023 | Increasing accessibility of data collection tools through technology and multi-platform processes. This includes tools such as photo-sharing software, virtual formats, audio recorders, and accessible materials. |
| 17. | Bard | 2021 | Utilizing accessible data collection devices and providing appropriate accommodations for pwd. |
| 18. | FDA (1) | 2023 | Accessible and supportive locations and tools for data collection. This includes providing alternative locations for data collection, utilizing accessible digital health tools and providing adequate transportation |
| 19 | National Academy of Sciences | 2022 | Building partnership, flexibility in data collection, input from community representatives, and adequate time and funding. |
| 20 | National Council on Disability | 2024 | Recommendation for disability to be collected on various demographic data sources. This includes adding demographic data such as disability in various federal requirements/agencies, providing flexibility in data collection protocols such as “considering frequency of planned visits and physical accessibility of trial sites.” |
| 21 | National Federation of the Blind | n.d. | Increasing data collection requirements, utilizing person-centered holistic data collection methods, and providing accommodations and tools. |
| 22 | Routen | 2022 | More inclusive demographic characteristics requirements. Collect data on social experiences to better understand causes of disparities, rather than relying on demographics as proxy |
| 23 | Petersen | 2023 | More structured data collection, including pwd in data collection process, and more transparent data governance. |
| 24 | Carneiro | 2025 | Communicate in accessible formats, such as plain language, videos or images, |
| 25 | Biggs | 2024 | Use multiple data collection methods including remote data collection methods, flexibility and accessible data collection materials. Document information alongside participants. Be flexible to reduce patient burden, such as allow for participants to be late. |
| 26 | Banas | 2019 | Provide accommodations (especially to people with cognitive disabilities), communicate with participants clearly. |
| 27 | Cockburn | 2024 | Employ possible accommodations and resources to promote access. Work to reduce underreporting of disability. Allow participants to select multiple types of disability or impairments, including episodic disability. |
| 28 | Ouellette | 2019 | Adequate accommodation (i.e. interpreters and assistive technology), time, and clear communication. Inaccessible medical equipment may contribute to exclusion of PWD. |
| 29 | Bradley | 2021 | Collect data on PWD in big data and longitudinal studies |

**Supplemental Table 7: Data Analysis Recommendations**

| Number | First Author | Year | Recommendation (Summary) |
| --- | --- | --- | --- |
| 1. | Janevic | 2022 | Interpreting data within context of diversity framework, utilizing data analysis techniques which accommodate intersectionality, and leveraging sample diversity in secondary data analysis. Include qualitative or mixed-methods approaches to capture intersectional experiences and contextual nuance. |
| 2. | Wang | 2023 | Recommendations for inclusion of pwd in synthesis and analysis of data. This includes engaging pwd and their families as part of the research team. |
| 3. | Frankena | 2019 | Providing training and engaging in discussions with team members about creative means for data analysis. |
| 4. | Witham | 2020 | Inclusive and accessible data analysis plans/protocols for pwd.  Analysis plans should include prespecified subgroup analyses to explore differential effects across under-served populations, including people with disabilities. |
| 5. | MRCT Center | 2023 | Implementing universal design in all aspects of clinical research from development, recruitment, data collection, data analysis, reporting and dissemination, and IRB/ethical concerns. This includes planning for subgroup data analysis to determine if metrics may need to be revised for more accurate data collection on pwd. |
| 6. | University of Washington | n.d. | Continual assessment of accessibility and inclusivity during analysis in order to make sure any exclusion of pwd is properly justified. This includes referring to guidance from the Accessibility by Design Toolkit. |
| 7 | Biggs | 2024 | Complete sub-group analysis, regardless of if powered. Evaluate participant demographics during studies to check for diverse representation. |

**Supplemental Table 8: Reporting and Dissemination Recommendations**

| Number | First Author | Year | Recommendation (Summary) |
| --- | --- | --- | --- |
| 1. | Janevic | 2022 | Engaging community organizations and advisory boards on appropriate reporting channels as well as providing training for possible advocacy utilization. This includes making sure the results are being disseminated in accessible manner (i.e. plain language with visuals). Additionally, developing community-facing dissemination products using accessible language and formats, and budgeting for translation and design. Reporting disaggregated findings for disability subgroups when possible and contextualize disparities using structural frameworks. Acknowledging intersectionality in interpreting results and avoid framing minoritized groups in deficit-oriented terms. Sharing preliminary findings with participants and community partners in plain language through newsletters or events. |
| 2. | Diemer | 2022 | Recommendations for considering implications/interpretations of possible results as well as training for supporting pwd. |
| 3. | St. John | 2022 | Developing inclusive dissemination strategies. This includes stakeholder engagement in the reporting and dissemination process as well as ensuring the accessibility of dissemination strategy. Use plain language and alternative dissemination formats (e.g., newsletters, videos, social media posts, summaries of papers) to increase accessibility. |
| 4. | Frankena | 2019 | Partnerships and clear communication between pwd and researchers, adequate accessibility (i.e. accessible abstract), and discussions on non-scientific publications and authorship decisions for pwd in the research team. |
| 5. | Witham | 2020 | Inclusive dissemination plan with community/stakeholder consultation with pwd. This includes incorporation of feedback, debate, and engagement with community/stakeholders. |
| 6. | MRCT Center | 2023 | Implementing universal design in all aspects of clinical research from development, recruitment, data collection, data analysis, reporting and dissemination, and IRB/ethical concerns. This includes using clear communication and accessible and health literate formats for disseminating study results to participants and the community. |
| 7. | Thompson | 2020 | Protecting the identity of participants while also reporting and disseminating the results in an accessible manner for the target audience. This includes utilizing clear communication in multiple formats, partnering with community stakeholders, and providing strategies for protecting the privacy of children with disabilities during dissemination. |
| 8. | University of Washington | n.d. | Providing aggregate trial results back to the participant community and providing avenues of participant feedback for effective reporting and dissemination and for better guidance on future research studies. This includes referring to guidance from the Accessibility by Design Toolkit. |
| 9. | Northwestern University Institutional Review Board Office | n.d. | Including pwd in the promotion, communication and identification to address results and barriers to research. This includes providing accessible communication materials for “inclusive reading and mental processing”. |
| 10 | National Academy of Sciences | 2022 | Increasing accessibility and transparency for results. This includes providing and updating a data dashboard and standardizing trial characteristics so they can be “easily disaggregated, exported, and analyzed by the public”. |
| 11 | National Council on Disability | 2024 | All reporting/dissemination materials to be accessible (“digitally accessible and section 508 Compliant”. |
| 12 | FDA (3) | 2023 | Proper data sharing and data return while also involving pwd in the reporting/dissemination process. This includes guidance on protecting privacy of participants, “clearly explaining the broader context of the study’s purpose and outcomes, transparently and accurately portraying the study results, and sharing demographic information to highlight the importance of representative clinical research” and using plain language and visual aids for more accessible report dissemination. |
| 13 | Rutta | 2024 | Recommendations for regular reporting requirements with publicly available data on study progress towards diversity plans. |
| 14 | Carneiro | 2025 | Use “accessible formats—for example, easy-to-read leaflets, videos, or social media posts.” |
| 15 | Kolbe | 2024 | Improve requirements by federal agencies and funding organizations for transparent and accountable data reporting. |
| 16 | Biggs | 2024 | Provide accessible materials for dissemination, such as with translations, clear language, and involvement of pwd in the development of dissemination plans. Share results in community settings. |
| 17 | Banas | 2019 | Share findings in multiple accessible formats for PWD |

**Supplemental Table 9: IRB/Ethics Recommendations**

| Number | First Author | Year | Recommendation (Summary) |
| --- | --- | --- | --- |
| 1. | Meierer | 2022 | Inclusion of the disability community, self-advocacy, combating the stereotypes which may be present in research ethics committee members. Adopting precise/up-to-date terminology for referring to participants with ID in ethical guidelines. IRBs should carefully balance protection from potential abuse with the necessity for fair representation and inclusion of individuals with intellectual disabilities in research. |
| 2. | McDonald | 2022 | Building flexibility and creativity in the research process to combat exclusion of pwd in clinical trials. |
| 3. | MacNeil | 2024 | Promoting a balance between participant protection and inclusion, in line with principles of equitable research access and the right to benefit from research. Educate researchers and gatekeepers about their ethical obligations not just to protect, but also to empower and include children with intellectual disabilities. This includes including pwd with “opportunity to ask questions and share their thoughts supports their ability to make decisions that affect them, respects their role in the research process, and allows them to be taken seriously; all of which help engage the child in the research” |
| 4. | St. John | 2022 | Partnership and trust building with pwd as part of the research team. Avoid blanket exclusions based on intellectual disability by shifting from protectionist to inclusive practices that still ensure participant safety. |
| 5. | Shariq | 2023 | Considering guidance by United nations Convention on the Rights of Persons with Disabilities and World Medical Association on inclusion/exclusion of pwd in clinical research. Partnership and including pwd in research process. |
| 6. | Raskoff | 2023 | Institutional/federal regulations/guidance for ethical research with pwd. This includes including pwd in ethical/advisory committees, accessible study material, adequate time/risk assessment, etc. |
| 7. | Deshpande | 2020 | Including pwd in Institutional Ethics Committees (IEC), providing adequate training fo IECs, and guidance surrounding consent (i.e. possibility of conflicting interests between participants and surrogates). |
| 8. | Frankena | 2019 | Recommendations for accessibility of pwd in research by ethical committees and journal editors. Promoting ethical awareness throughout the research process, including respecting diverse cultural and representational experiences. |
| 9. | Dakic | 2020 | Considerations around risk assessment for inclusion of pwd in research carried out in partnership with the community and pwd. This includes involving pwd and communities as co-researchers and in IRBs as well as guidance for consent processes. |
| 10. | Witham | 2020 | Inclusivity/accessibility requirements for Ethics Committees and funders, such as ensuring accessibility of materials, justification for exclusion criteria and encouraging creative methods for “consenting, recruiting and retaining participants in research studies”. |
| 11. | MRCT Center | 2023 | Implementing universal design in all aspects of clinical research from development, recruitment, data collection, data analysis, reporting and dissemination, and IRB/ethical concerns. This includes engaging pwd as IRB members, providing adequate time, trainings, and funding for researchers and IRBs to design and review study protocol, and guidance for IRBS when planning institutional policies about supported decision-making. |
| 12. | Shepherd (1) | 2020 | Ethical recruitment, enrollment, and consent process for clinical trial participation. This includes recommendations for prospective appointment to surrogates and use of ARDs. In addition, recommendations for IRBs to require appropriate exclusion justification when approving research and funding. |
| 13. | Thompson | 2020 | Recommendations for protecting the identity of participants and ethical considerations around including pwd in research. |
| 14. | Shepherd (2) | 2020 | Increased requirement for clarity, explicitness, and accuracy when reviewing and approving research with pwd by Research Ethic Committees (RECs). |
| 15. | University of Washington | n.d. | Recommendations for IRB/Ethical Review Boards on how to increase inclusivity for pwd in research including utilizing accessible design, accessible material, providing adequate time for consent and study activities,  ethical consent process, etc. |
| 16. | DeCormierPlosky | 2022 | Including clear requirements for explicit ethical justification for exclusion criteria, accommodations, alteration, and legal requirements. Recommend that IRBs and sponsors review eligibility criteria to identify overly broad exclusion language. |
| 17. | McDonald | 2023 | Recommendations for ethical consent, accommodation, and clear justified exclusion criteria for pwd. This includes adequate anti-ableist training. |
| 18 | Cunningham | 2025 | Including pwd in ethics review boards and incorporating community advocacy into ethics committees and funding bodies. |
| 19. | Friesen | 2023 | Building reflective question in IRB review process to guide obligations to protect vs to include pwd, requiring justification for exclusion criteria and building flexibility and accessibility into study protocols. This includes input from pwd and disability community. |
| 20 | WHO | 2022 | Including pwd in ethical committees and advisory boards. |
| 21 | Wickremsinhe | 2023 | Inclusion of pwd based on an ethical imperative based on 2 frameworks- Council for International Organizations of Medical Sciences (CIOMS) and CRPD. This includes providing justification for exclusion, guidance for ethical consent process, and the ethical principle of non-discrimination. |
| 22 | Bard | 2021 | Legal ethical mandates towards requiring accessibility through accommodations in research trials for pwd. This includes ethical and legal considerations such as ADA mandated accessible medical equipment in research and appropriate requirements for accommodations by sponsors/funders. |
| 23 | UCSF | 2023 | Recommendations for IRB requirements about consent to make it more accessible and inclusive for pwd. This includes IRB considerations for ethical consent process, adequate safeguards and educational training. |
| 24 | Northwestern University Institutional Review Board Office | n.d. | Inclusion of pwd in IRB committees and reviewing policy to encourage the participation of pwd in research. |
| 25 | National Academy of Sciences | 2022 | Increased requirements by IRBs, editors, funders, and publishers on representativeness of pwd in trials and studies. |
| 26 | ASH Clinical News | 2021 | Recommendations for ethical consent protocol. This includes providing adequate time and engagement with families/guardians of the participant and ethical consultation to “help determine a patient's capacity to consent or clarify a gray area in which participation in clinical research may be inappropriate” |
| 27 | National Council on Disability | 2024 | Increased IRB requirements and justifications when approving and funding studies. Utilization of plain language and accessible materials as well as including pwd on advisory boards. |
| 28 | University of Michigan | 2023 | Accessibility and accommodation requirements for IRB approval. This includes providing accessible study design to be evaluated by the IRB. |
| 29. | Silverman | 2022 | Recommends IRBs understand supported-decision making laws and require study protocols to address and comply with them |
| 30 | FDA (3) | 2023 | Involving pwd in IRB/ethics committees as well as in the research design/planning phase. |
| 31 | Rutta | 2024 | Partnering with pwd and other experts to increase accessibility for pwd. This includes requirements for adequate budgets, accessible materials, and inclusive logistical protocols for pwd. |
| 32 | Dubreuil | 2024 | Including pwd on IRB and Ethical advisory boards and providing proper training/skill development for inclusive ethical review. |
| 33 | Carneiro | 2025 | Involve people with intellectual disabilities and their advocates in research ethics committees and provide appropriate accommodations to them. |
| 34 | Leigh | 2025 | Recommendations for including pwd in the planning and development stages of research. This includes partnering with stakeholders, community organizations, and the creation of regulatory guidelines. This includes complying with all the accessibility and inclusivity requirements of various ethical frameworks and ethical review boards. |
| 35 | Banas | 2019 | Educate and collaborate with ethics boards on including PWD to studies. |
| 36 | Ouellette | 2019 | IRBs have historically been overprotective and may now move to provide adequate accommodations and support of universal research design. This includes guidance on ethical consent protocol and requiring clear justification for exclusion of pwd in research. |

**Supplemental Table 10: Cross-cutting Themes Across Domain**

| Number | First Author | Year | Recommendation (Summary) |
| --- | --- | --- | --- |
| People with Disabilities as researchers and stakeholders | | | |
| 1. | Camanni | 2023 | Including pwd as active partners and stakeholders through accommodations and accessible research protocols. |
| 2. | Thurm | 2022 | Increasing community partnership and accessibility for pwd. This includes increased flexibility to promote research engagement, increased training, involvement of community liaisons, etc. |
| 3. | Heath | 2022 | Engaging in open communication, integrating pwd into the design and planning of the study, etc. Build relationships based on trust, respect, transparency, and shared decision-making between researchers and disability community members. Recognize and value the diverse expertise that people with disabilities bring, fostering open communication and co-designed research approaches. |
| 4. | St. John | 2022 | Building partnership and including pwd in planning, development, and implementation of research and dissemination of results. Including self-advocates with intellectual disability as co-researchers in all stages, including conceptualization, data interpretation, and dissemination. |
| 5. | Wang | 2023 | Involving pwd on research team and building partnership and trust with them and their families. This includes engaging pwd and their families, providing adequate time and accessible materials during the research process. Ensure stakeholders receive feedback on their contributions and are acknowledged in presentations or publications. |
| 6. | Mintz | 2022 | Inclusion of pwd as part of the research team and training for researchers. |
| 7. | Frankena | 2019 | Involving people with intellectual disabilities in all stages of research—from conceptualization to dissemination—according to their preferences and capacities. Providing tailored support, mentoring, and training to enable meaningful participation. |
| 8. | Nguyen | 2019 | Providing clarity on roles and responsibilities for team members and building partnership and trust with pwd in research. Providing adequate time, funding, and accessible materials/protocols for inclusive research. |
| 9. | McDonald | 2023 | Recommendations for community partnership and engagement in all aspects of research process. This includes community-supported consent protocols, anti-ableist frameworks, presuming competence with supported decision-making, and community engagement to build trust. |
| 10 | Cunningham | 2025 | Involving community partnership in the planning, recruitment, and implementation of research through use of guidance by the Accessibility by Design Toolkit.” |
| 11 | WHO | 2022 | Partnering with pwd in training, increased collaboration with pwd as partners and consultants, and partnership with community/institutions. |
| 12. | Sadler | 2023 | Involving pwd as advisors and contributors during the research process. This includes including people with IDD in design and administration of research to build trust and respect while ensuring accessibility, flexibility and accommodations. |
| 13 | Northwestern University Institutional Review Board Office | n.d. | Including pwd as partners and co-researchers. Partnering with the community and including the perspectives and feedback of pwd in the design and implementation of research, |
| 14 | Sakuma | 2024 | Utilization of community input through participatory research approach, legislative action on inclusion of pwd in clinical trials and providing trainings for stakeholders in the design of disability guidelines in research. |
| 15 | Rutta | 2024 | Partnership with pwd in all phases of research design. This includes community engagement and cultural competency training for investigators, building trust, and “encouraging sponsors to partner with community organizations, engage with local leaders, and tailor their outreach efforts to the needs of specific populations”. |
| 16 | Dubreuil | 2024 | Partnering with pwd in study design, recruitment, data collection and IRB/advisory roles. |
| 17 | Berg | 2024 | Seek out and incorporate youth and caregiver feedback on planning, development, study materials, and consent phases. |
| 18 | Biggs | 2024 | Partner with community organizations and reduce the hierarchy between researchers and participants through sharing power in all stages of research. |
| 19 | Banas | 2019 | Employ and support PWDs’ careers in research studies, such as via assistive technology. Partner with the disability community to develop the study. |
| 20 | Cockburn | 2024 | Include pwd in the process of selecting questions to ask about disability status and providing team members with proper training, time, and resources for adequate inclusion of pwd in research. |
| 21 | Bradley | 2021 | Involve disability in engineering and product design education. Involve PWD in scientific advancement and product design that affect their lives. |
| Consent | | | |
| 1. | Meierer | 2022 | Avoid excluding individuals with intellectual disabilities based solely on assumptions about their ability to consent; carefully assess each participant's actual decision-making capability.  Actively seek and document assent from participants unable to fully consent, clearly respecting dissent at all times by allowing withdrawal from the study. Use personalized, accessible, and comprehensible communication methods tailored to the cognitive and communicative abilities of individuals with ID to ensure informed decision-making about participation. Provide ample time for participants with intellectual disabilities and their support networks to discuss and consider participation thoroughly. |
| 2. | McDonald | 2024 | Providing education/trainings, justification for exclusion criteria, and developing accessibility requirements/protocols for individuals “who do not demonstrate consent capacity). Presenting study procedures slowly, with time for clarifications and repeating explanations. Developing combined consent-assent forms where guardianship is involved, and prioritize the individual's understanding and willingness even under guardianship. Importance of not assuming adults with ID have a guardian, and probing to verify guardianship status. |
| 3. | McDonald | 2022 | Ethical consent including presumption of competence of prospective participants, proper eligibility criteria rationale, and accommodations for pwd and LAR in the consent process. Develop cognitively accessible consent materials and protocols to enhance understanding. Apply supported decision-making strategies to allow adults with intellectual disability to participate in consent processes with appropriate aids and guidance. Ensure that assessments of consent capacity are appropriate to the individual and avoid reliance on default assumptions based on disability diagnosis. |
| 4. | Piantedosi | 2023 | Informal communication ability assessments and personalized approaches, written consent supplemented with plain language explanations and visual aids to ensure comprehension. Allocating time and resources to support informed consent and comfortable participation environments. |
| 5. | MacNeil | 2024 | Recommendations to obtain ethical consent including engaging child with disability in the assent/dissent process while allowing participant “with an opportunity to ask questions and share their thoughts supports their ability to make decisions that affect them, respects their role in the research process, and allows them to be taken seriously; all of which help engage the child in the research”. |
| 6. | Thurm | 2022 | Recommendations for adapting existing consent materials and implementing measures to allow for ethical inclusion and consent for pwd. |
| 7. | Deckler | 2022 | Guidance on protocol for obtaining ethical consent including division of labor and responsibilities within the research team and accessible consent materials. |
| 8. | St. John | 2022 | Accessible consent process including using plain and succinct language, including simple images/diagrams, utilizing universal accessibility and including stakeholders int he consent process. |
| 9. | Shariq | 2023 | Improving accessibility for the consent process including “tailoring of consent processes, the use of decision aids to improve accessibility, and revisiting consent after data collection”. This includes utilizing clear communication between participant and research team, accessible consent materials, including advocates and community partners, and empowering participants to contribute to decision making. |
| 10. | Raskoff | 2023 | Ensuring “that the consenting individual understands how to withdraw consent and that they can do so at any point. Researchers should continuously assess whether the planned number of rounds of noxious stimuli are tolerated by participants and necessary to answer their research questions and should be prepared to modify their experimental design if they are not.” Recognize that children and adolescents with severe ID may express dissent in atypical, non-verbal ways. Ensure caretakers are present during study participation to identify and advocate for signs of distress, discomfort, or resistance. Obtain informed permission from legally authorized representatives (LARs). Ensure representatives understand withdrawal rights and study risks. |
| 11. | Russell | 2023 | Ethical consent including clear justification of inclusion/exclusion of adults who ”lack capacity”. This includes involving stakeholders (e.g., legal representatives such as family members or professional advocates) in consent process. |
| 12. | Deshpande | 2020 | Assess decisional capacity specific to the research task and ensure participants understand procedures, risks, and benefits; mental illness alone does not imply incapacity. Modify consent procedures (e.g., simplify information, provide support, assess comprehension) to accommodate those with impaired capacity. Avoid enrolling institutionalized individuals without decisional capacity unless participation offers a substantial therapeutic benefit unavailable elsewhere. |
| 13. | Dakic | 2020 | Establishing clear guidance on the consent process for pwd. This includes recommendations for the use of UK Mental Act of 2005 guidance on consent and utilizing the view of consent as an ongoing process as opposed to a one-time event. |
| 14 | MRCT Center | 2023 | Implementing universal design in all aspects of clinical research from development, recruitment, data collection, data analysis, reporting and dissemination, and IRB/ethical concerns. This includes taking trainings, and using plain language, providing adequate time, support, accommodations, and modifications for the informed consent process, |
| 15. | Shepherd (1) | 2020 | Recommendations for ethical consent including prospectively nominating a surrogate for research and creating ARDs. |
| 16. | Shepherd (2) | 2020 | Obtaining ethical consent, explicitly stating justification for the exclusion of pwd in clinical trials, includingr greater active involvement of research funding organizations to scrutinize the justifications behind exclusion. |
| 17. | University of Washington | n.d. | Increased accommodation and accessibility during the consent process. This includes accommodation and use of LAR in the consent process. |
| 18. | DeCormierPlosky | 2022 | Recommendation for inclusive consent through accessible materials and ethical considerations. |
| 19. | Wickremsinhe | 2023 | Recommendations for assessing the capacity to consent and need for substitute decision making for people with psychosocial disabilities. |
| 20. | FDA (1) | 2023 | Assumption of competence for consent in clinical study participation unless otherwise determined as well as best practices for guardians/legal representatives for consent. Supports cognitive assessments and collaborations with psychologists to determine ability to consent. |
| 21. | UCSF | 2023 | Recommendations for IRB requirements for including assessing decisional capacity and need for surrogate consent for enrolling participants with cognitive impairments based on study risk. This includes requirement for additional safeguards, using an independent monitor, educational techniques, use of waiting periods, and surrogate permission for participants “who do not have decisional capacity to consent for themselves” |
| 22 | FDA (2) | 2023 | Recommendations for ethical informed consent with guidance on accessibility and provision of reasonable modifications and auxiliary aids and services, use of an independent qualified professional, additional time for decision-making, using methods to enhance consent capacity, involving an LAR. |
| 23 | ASH Clinical News | 2021 | Ethical consent protocol including ethical consultation with pwd and the community. This includes involving pwd, parent, and guardians as proxy for patients, ethical consultation to help “determine a patient's capacity to consent or clarify a gray area in which participation in clinical research may be inappropriate”. |
| 24 | National Council on Disability | 2024 | Existing guidelines and exclusion justifications for pwd in obtaining informed consent. This includes providing adequate accommodations for study materials, additional time, caregiver support, and auditory presentation for participants with impaired consent capacity. |
| 25 | University of Michigan | 2023 | Ethical informed consent protocol including providing adequate time and accessible materials during the consent process. |
| 26 | Silverman | 2022 | Recommendations to provide, but not require, decision-related accommodations in consent process. This includes accessible capacity assessment, flexible consent protocol, involvement of surrogates (if necessary), and inclusion of pwd in the” capacity assessments, consent process, and other research procedures |
| 27 | FDA (3) | 2023 | Ethical consent while not presuming that pwd cannot consent. This includes providing accommodations, “facilitating supported decision-making, and ensuring that participants have the information they need to consent”. |
| 28 | Carneiro | 2025 | Use accessible consent materials/formats including usage of plain language and guidance on legally authorized representatives (LARs). |
| 29 | Leigh | 2025 | Inclusive consent process including utilization of plain and culturally relevant language, accessible consent materials, inclusion of parent/community stakeholders in consent process. |
| 30 | Kolbe | 2024 | FDA re-defined their understanding of vulnerable populations relating to disability from “handicapped, or mentally disabled persons” to “persons with impaired decision-making capacity.” |
| 31 | Agaronnik | 2025 | Provide accessible materials, auxiliary aids, and materials, and options for supported decision-making for participants. |
| 32 | Biggs | 2024 | Collect consent in stages, check for participant understanding, provide accommodations such as supporters for those with low literacy, provide multiple modalities for consent such as e-consent, use simple language |
| 33 | Ouellette | 2019 | Researchers and IRBs should provide adequate accessibility and accommodation during the consent process. This includes clear language, interpreters, and assistive technology. Do not assume people with intellectual disabilities cannot consent. |
| 34 | Bradley | 2021 | Clear communication and use of accommodations/assistive technology “to ensure that they are neither exploited nor excluded from scientific studies” especially people with intellectual disabilities. |

# **Supplemental Table 11: List of Relevant Documents**

| Number | First Author | Year | Disability (Type) | Country or region in which the policy or guidance originates | Country or region in which the policy or guidance is aimed towards | Search Strategy | Target Audience |
| --- | --- | --- | --- | --- | --- | --- | --- |
| 1 | Kushalnagar, Poorna | 2023 | Deaf/Hard of Hearing | United States | Undefined | Peer-Reviewed Databases | Academics/Researchers |
| 2 | Camanni, Guido | 2023 | Any disability/undefined ; physical impairment, cognitive impairment, behavioural or psychiatric  disorders, communication and language impairment | Undefined; Scoping review, so literature from many places. Although main author is from Italy | International | Peer-Reviewed Databases | Academics/Researchers |
| 3 | Meierer,  Klara | 2022 | ID/IDD | taly | International | Peer-Reviewed Databases | Academics/Researchers |
| 4 | McDonald, Katherine | 2024 | ID/IDD | United States | United States | Peer-Reviewed Databases | Academics/Researchers |
| 5 | McDonald, Katherine | 2022 | ID/IDD | United States | United States | Peer-Reviewed Databases | Academics/Researchers |
| 6 | Piantedosi, Diana | 2023 | ID/IDD | International | International | Peer-Reviewed Databases | Academics/Researchers; Policy Makers |
| 7 | MacNeil, Morgan | 2024 | ID/IDD | Canada | Undefined | Peer-Reviewed Databases | Academics/Researchers |
| 8 | Janevic, Mary | 2022 | Any disability/undefined | United States | Undefined | Peer-Reviewed Databases | Academics/Researchers |
| 9 | Thurm, Audrey | 2022 | ID/IDD; ASD | United States | Undefined | Peer-Reviewed Databases | Academics/Researchers |
| 10 | Diemer, Maire | 2022 | Autism | United States | United States | Peer-Reviewed Databases | Academics/Researchers; Clinicians |
| 11 | Heath, Gregory | 2022 | Any disability/undefined ; People with Spinal Cord Injury  People with stroke  People with Parkinson Disease,  People with Cerebral Palsy | United States | International | Peer-Reviewed Databases | Academics/Researchers; Policy Makers; Funding agencies/organizations |
| 12 | Deckler, Elizabeth | 2022 | Schizophrenia | United States | Undefined | Peer-Reviewed Databases | Academics/Researchers |
| 13 | St. John, Brittany | 2022 | ID/IDD | United States | United States | Peer-Reviewed Databases | Academics/Researchers |
| 14 | Wang, Lucy | 2023 | Neurodisability | Canada | Undefined | Peer-Reviewed Databases | Academics/Researchers |
| 15 | Shariq, Sameed | 2023 | Any disability/undefined | United Kingdom | Undefined | Peer-Reviewed Databases | Academics/Researchers |
| 16 | Raskoff, Sarah | 2023 | ID/IDD; Severe Intellectual disability | United States; United Kingdom | Undefined | Peer-Reviewed Databases | Academics/Researchers |
| 17 | Russell, Amy | 2023 | Any disability/undefined ; ID/IDD; Deaf/Hard of Hearing; Vision; Communication disabilities, autism | United Kingdom | United Kingdom | Peer-Reviewed Databases | Academics/Researchers |
| 18 | Deshpande, Smita | 2020 | Mental Health | India | India | Peer-Reviewed Databases | Academics/Researchers |
| 19 | Mintz, Kevin | 2020 | Physical and Sensory Disabilities | United States | United States | Peer-Reviewed Databases | Academics/Researchers; Therapists |
| 20 | Frankena, T.K. | 2019 | ID/IDD | International | Undefined | Peer-Reviewed Databases | Academics/Researchers |
| 21 | Nguyen, Tram | 2019 | Any disability/undefined ; Youth with disabilities | Undefined | Undefined | Peer-Reviewed Databases | Academics/Researchers |
| 22 | Dakic, Tea | 2020 | Mental Health | Montenegro | Montenegro | Peer-Reviewed Databases | Academics/Researchers; Policy Makers |
| 23 | Witham, Miles | 2020 | Any disability/undefined | United Kingdom | Undefined | Peer-Reviewed Databases | Academics/Researchers; Policy Makers |
| 24 | MRCT Center | 2023 | Any disability/undefined | United States | Undefined | Grey Literature | Academics/Researchers; Advocates; ethics committees/IRBs, research participants, family, allies |
| 25 | Shepherd, Victoria (1) | 2020 | Any disability/undefined; learning disabilities, cognitive impairments | United Kingdom | Undefined | Grey Literature | Academics/Researchers; Policy Makers |
| 26 | Thompson, Stephen | 2020 | Children with disabilities | United Kingdom | International | Grey Literature | Academics/Researchers; Policy Makers; Advocates |
| 27 | Shepherd, Victoria (2) | 2020 | Any disability/undefined; ID/IDD; Mental Health ; Cognitive impairment, autism, learning disabilities | United Kingdom | Undefined | Grey Literature | Academics/Researchers; Policy Makers |
| 28 | University of Washington | n.d. | Any disability/undefined | United States | United States (Organizational/Institutional) | Grey Literature | Academics/Researchers; IRB |
| 29 | DeCormierPlosky, Willyanne | 2022 | Any disability/undefined | United States | Undefined | Grey Literature | Academics/Researchers |
| 30 | McDonald, Katherine | 2023 | ID/IDD | United States | Undefined | Grey Literature | Academics/Researchers |
| 31 | Cunningham, Shauna | 2025 | Any disability/undefined | United Kingdom | Undefined | Google Scholar | Academics/Researchers |
| 32 | Friesen, Phoebe | 2023 | ID/IDD; Mental Health | United States | United States | Grey Literature | Academics/Researchers; IRB |
| 33 | World Health Organization (WHO) | 2022 | Any disability/undefined | International WHO | International | Grey Literature | Academics/Researchers; Policy Makers; Advocates |
| 34 | Sadler,  Tonie | 2023 | ID/IDD | United States | Undefined | Grey Literature | Academics/Researchers |
| 35 | Wickremsinhe, Marisha | 2023 | psychosocial disabilities | Australia | International; 26 African countries Malaysia and Peru. | Grey Literature | Academics/Researchers; Policy Makers |
| 36 | Bard, Jennifer | 2021 | Any disability/undefined | United States | United States | Grey Literature | Academics/Researchers |
| 37 | FDA (1) | 2023 | ID/IDD; Autism, Down Syndrome | United States | United States | Grey Literature | FDA Commissioner |
| 38 | Office for Civil Rights (OCR) | 2023 | Any disability/undefined | United States | United States | Grey Literature | Undefined |
| 39 | University of California San Francisco (UCSF) | 2023 | Cognitive Impairments | United States | United States (Organizational/Institutional) | Grey Literature | Academics/Researchers |
| 40 | FDA (2) | 2023 | physical or sensory disabilities, adults with impaired consent capacity | United States | United States | Grey Literature | Academics/Researchers; IRBs, Clinical Investigators, and Sponsors |
| 41 | Chen, Szu-Wei | 2024 | Any disability/undefined | United States | United States | Grey Literature | Academics/Researchers |
| 42 | Northwestern University Institutional Review Board Office | n.d. | Any disability/undefined | United States | United States (Organizational/Institutional) | Grey Literature | Academics/Researchers |
| 43 | Andrews, Emma | 2020 | Any disability/undefined | United States | Undefined | Grey Literature | Academics/Researchers |
| 44 | National Academy of Sciences | 2022 | Any disability/undefined | United States | United States | Grey Literature | Academics/Researchers |
| 45 | Sakuma, Yoshiko | 2024 | Any disability/undefined | United Kingdom | United Kingdom | Grey Literature | Academics/Researchers |
| 46 | ASH Clinical News | 2021 | ID/IDD | United States | Undefined | Grey Literature | Academics/Researchers |
| 47 | National Council on Disability | 2024 | Any disability/undefined | United States | United States | Grey Literature | Academics/Researchers; Policy Makers; Healthcare practitioners |
| 48 | University of Michigan | 2023 | Deaf/Hard of Hearing; Vision | United States | United States (Organizational/Institutional) | Grey Literature | Academics/Researchers |
| 49 | Silverman, Benjamin | 2022 | Any disability/undefined | United States | Undefined | Grey Literature | Academics/Researchers |
| 50 | FDA (3) | 2023 | Any disability/undefined; ID/IDD, mental illness | United States | United States | Grey Literature | Academics/Researchers; Advocates; Drugs and medical device sponsors |
| 51 | National Federation of the Blind | n.d. | Any disability/undefined | United States | Undefined | Grey Literature | Academics/Researchers |
| 52 | Routen, Ash | 2022 | Any disability/undefined | United Kingdom | United Kingdom | Grey Literature | Academics/Researchers |
| 53 | Petersen, Carolyn | 2023 | Any disability/undefined | United States | United States | Grey Literature | Academics/Researchers |
| 54 | Rutta, Randall | 2024 | Any disability/undefined | United States | United States | Grey Literature | Academics/Researchers; Policy Makers |
| 55 | Lamontagne, Marie-Eve | 2021 | traumatic brain injury | Canada | Undefined | Grey Literature | Academics/Researchers |
| 56 | Dubreuil, Maureen | 2024 | Patients with spondyloarthritis | United States | Undefined | Grey Literature | Academics/Researchers |
| 57 | Mishra, Shiva Raj | 2025 | Any disability/undefined | Australia | Undefined | Google Scholar | Academics/Researchers; Policy Makers |
| 58 | Carneiro, Lara | 2025 | ID/IDD | International | International; Europe | Google Scholar | Academics/Researchers |
| 59 | Leigh, Stephanie | 2025 | Any disability/undefined; Chronic Illness; Mental Health ; physical or mental disabilities, cognitive impairments, differently abled, neurological disabilities | South Africa | International | Google Scholar | Academics/Researchers; Policy Makers |
| 60 | Brathwaite, Justin | 2024 | Any disability/undefined | United States | Undefined | Google Scholar | Academics/Researchers; industry leaders |
| 61 | Berg, K.L. | 2024 | ID/IDD | United States | Undefined | Google Scholar | Academics/Researchers; Clinicians |
| 62 | Kolbe, Allison | 2024 | Any disability/undefined | United States | United States | Google Scholar | Academics/Researchers |
| 63 | Agaronnik, Nicole | 2025 | psychiatric and cognitive disabilities | United States | United States | Google Scholar | Academics/Researchers |
| 64 | Biggs, K | 2024 | Any disability/undefined | United Kingdom | Undefined | Google Scholar | Academics/Researchers |
| 65 | Banas, Jennifer | 2019 | Any disability/undefined | United States | Undefined | Grey Literature | Academics/Researchers |
| 66 | Schwartz, Jaclyn | 2021 | Any disability/undefined; Deaf/Hard of Hearing; Vision; Mobility; Cognitive impairment, Caregiver assistance, MEMS | United States | Undefined | Grey Literature | Academics/Researchers |
| 67 | Cockburn, Lynn | 2024 | Any disability/undefined | Canada | Undefined | Grey Literature | Academics/Researchers |
| 68 | Ouellette, Alicia | 2019 | Any disability/undefined | United States | Undefined | Grey Literature | Academics/Researchers; Policy Makers |
| 69 | Bradley, Valerie | 2021 | Any disability/undefined | Undefined | Undefined | Grey Literature | Academics/Researchers; Policy Makers |
