# Supplementary material for: Equitable inclusion of people with disabilities in clinical trials: a scoping review
Source: BMJ Open. 2026 Feb 4;16(2):e108550. doi: 10.1136/bmjopen-2025-108550 (PMC12878309; doi:10.1136/bmjopen-2025-108550)
Supplement: Supplementary file 2 [file bmjopen-2025-108550.reviewer_comments.pdf]

PEER REVIEW HISTORY

BMJ Open publishes all reviews undertaken for accepted manuscripts. Reviewers are asked to complete a checklist review form and are provided with free text boxes to elaborate on their assessment. These free text comments are reproduced below.

ARTICLE DETAILS

Title (Provisional)

Equitable Inclusion of People with Disabilities in Clinical Trials: A Scoping Review

Authors

Jo, Grace; Castro, Franz; Cerilli, Caroline; Varadaraj, V; Rosman, Lori; Kamenov, Kaloyan; Barrett, Darryl; Swenor, B

VERSION 1 - REVIEW

**Reviewer**	**1**
**Name**	**Berens, John**
**Affiliation**	**Baylor College of Medicine**
**Date**	**17-Nov-2025**
**COI**	**None**

This manuscript addresses a very important topic: the equitable inclusion of persons with disabilities in clinical trial research. The scoping review performed here was an appropriate method to synthesize existing recommendations and was completed according to accepted standards (i.e., PRISMA guidelines).

The execution of this review was performed thoroughly and succinctly, and is nearly in publishable form in this reviewer's opinion.

The Introduction gives a broad overview of the relevant background; if space allows, I think it might be helpful to introduce/define the various domains of disability that are later discussed (i.e., cognitive, physical, mental health). It may also be useful to discuss specific barriers or considerations that are more relevant to these specific subgroups, though this level of detail may not be desired for this particularly manuscript that provides a broad overview. If so, it could be helpful to acknowledge that different types of disabilities may face different barriers and require unique interventions (i.e., one size may not fit all).

Otherwise, there are just two small areas for correction:

1) Results, #6, last line has a typo, please correct.

2) Discussion, paragraph 1: “The exclusion of people with Down syndrome from trials and studies related to Alzheimer’s disease.” This sentence is a fragment, please revise for flow and clarity.

**Reviewer**	**2**
**Name**	**Anil, Krithika**
**Affiliation**	**University of Plymouth**
**Date**	**03-Dec-2025**
**COI**	**None**

Firstly, it is incredibly positive the authors include advocates for the population the paper aims to support, i.e., people with disabilities. I commend the research team for this inclusion.

The inclusion of people with disabilities in clinical trials is crucial for the progress of science. As the authors have rightly stated, this population is often excluded due to practicalities and perceived barriers that are not grounded in scientific justification.

I believe this piece of work has potential to be important, but it does not address its goal stated at the end of the introduction: “The goal of this work is to synthesize the evidence to offer a practical reference to support the integration of disability inclusion and equity into the design and conduct of clinical trials”. While this work collates the common considerations reported by current literature on the inclusion of people with disabilities in clinical trials, it stops short of being a practical reference for research teams when designing clinical trials.

The current results and summary in Table 2 are broad; it would be difficult to use as a practical starting point for someone designing a clinical trial. For example, the domain “inclusive and universal study design” refers to high-level concepts such as “universal design” and “anti-ableist frameworks,” which are conceptually important but practically vague. It would strengthen the paper considerably if the authors could break these concepts down into more actionable components drawn directly from the included sources.

Several approaches could be used to address this. Perhaps the authors could map the collated recommendations onto the main stages of a clinical trial? Or organise the results into common areas of accessibility needs, such as communication, cognitive support, or environmental barriers? These approaches would not introduce new guidance or recommendations, but it may provide research teams with clearer points of entry when designing inclusive clinical trials for people with disabilities.

Another critique is that the methods do not mention how you developed the search strategy, e.g., what was your review question, and what search term framework did you use (PCC, etc.)? The inclusion criteria is also not clearly stated.

Also, please note that the PRISMA framework is a reporting guideline, it does not provide guidance on how to do a scoping review. Please correct the phrasing in the paper.

If the above can be addressed, this work would be an incredibly valuable resource.

VERSION 1 - AUTHOR RESPONSE

Reviewer: 1
Dr. John Berens, Baylor College of Medicine
Comments to the Author:

This manuscript addresses a very important topic: the equitable inclusion of persons with disabilities in clinical trial research. The scoping review performed here was an appropriate method to synthesize existing recommendations and was completed according to accepted standards (i.e., PRISMA guidelines).

The execution of this review was performed thoroughly and succinctly, and is nearly in publishable form in this reviewer's opinion.

The Introduction gives a broad overview of the relevant background; if space allows, I think it might be helpful to introduce/define the various domains of disability that are later discussed (i.e., cognitive, physical, mental health). It may also be useful to discuss specific barriers or considerations that are more relevant to these specific subgroups, though this level of detail may not be desired for this particularly manuscript that provides a broad overview. If so, it could be helpful to acknowledge that different types of disabilities may face different barriers and require unique interventions (i.e., one size may not fit all).

Thank you for this important comment.
We avoided applying a stringent definition of disability and instead we were interested in documenting how disability was defined in the manuscripts and materials reviewed. In the Methods, we state that we did not limit our search to a specific definition of disability. In paragraph 2 of the Results we state how the different documents in the scoping review defined disability.

> (Page 5 and 6, Lines 187 to 194)
> "Disability was defined in various ways in the included documents, reflecting both medical and social models. While the majority of documents did not explicitly define disability (n=50)[5,8,11,12,15-60], ten documents used more medicalized definitions that emphasize functional limitations, impairments, and clinical diagnostic criteria (i.e. Diagnostic and Statistical Manual of Mental Disorders and the International Classification of Diseases).[61-69] The remaining nine documents used social models of disability (i.e. WHO International Classification of Functioning, Disability and Health (ICF), the Washington Group on Disability Statistics, and the United Nations Conventions on the Rights of Persons with Disability).[10,70-77]"

We listed the different disability types in paragraph 1 of Results (people with intellectual and developmental disabilities (IDD), mobility disabilities, sensory disabilities, mental health conditions, and cognitive impairments). We added these disability types into the introduction when discussing common barriers to inclusion of people with disabilities in clinical trials.

> (Page 3, Lines 90 to 93)

“The broad diversity in the population (with various disability types including people with intellectual and developmental disabilities (IDD), mobility disabilities, sensory disabilities, mental health conditions, and cognitive impairments) and the variance in defining disability can exacerbate these mistaken assumptions.”

We very much agree with this reviewer that people with different types of disabilities may face different barriers that could necessitate specific interventions. This reviewer is correct that this aims to be a manuscript about the broad barriers and approaches that can be taken to move the needle. We have revised the Discussion to highlight that one size does not fit all and appreciate the reviewer pointing out the need for this statement.

(Page 9, Lines 337 to 339)
“It should be noted that this review focused on the common clinical trial barriers that people with disabilities face. However, people with different types of disabilities often face unique barriers that may necessitate specific interventions and approaches.”

Otherwise, there are just two small areas for correction:
1) Results, #6, last line has a typo, please correct.

We have fixed the typo. The last line now reads,

(Page 7, Line 262 to 266)
“Four documents highlighted the need to align review process with rights-based frameworks and legal mandates (i.e. the Americans with Disabilities Act or the United Nations Convention on the Rights of People with Disabilities) to reinforce ethical accountability and support ethical mandates for accessibility.[10,48,64,74]”

2) Discussion, paragraph 1: “The exclusion of people with Down syndrome from trials and studies related to Alzheimer’s disease.” This sentence is a fragment, please revise for flow and clarity.

This sentence fragment was removed for flow and clarity.

Reviewer: 2
Dr. Krithika Anil, University of Plymouth
Comments to the Author:

Firstly, it is incredibly positive the authors include advocates for the population the paper aims to support, i.e., people with disabilities. I commend the research team for this inclusion.

The inclusion of people with disabilities in clinical trials is crucial for the progress of science. As the authors have rightly stated, this population is often excluded due to practicalities and perceived barriers that are not grounded in scientific justification.

I believe this piece of work has potential to be important, but it does not address its goal stated at the end of the introduction: “The goal of this work is to synthesize the evidence to offer a practical reference to support the integration of disability inclusion and equity into the design and conduct of clinical trials”. While this work collates the common considerations reported by current literature on the inclusion of people with disabilities in clinical trials, it stops short of being a practical reference for research teams when designing clinical trials.

The current results and summary in Table 2 are broad; it would be difficult to use as a practical starting point for someone designing a clinical trial. For example, the domain “inclusive and universal study design” refers to high-level concepts such as “universal design” and “anti-ableist frameworks,” which are conceptually important but practically vague. It would strengthen the paper considerably if the authors could break these concepts down into more actionable components drawn directly from the included sources.

Several approaches could be used to address this. Perhaps the authors could map the collated recommendations onto the main stages of a clinical trial? Or organize the results into common areas of accessibility needs, such as communication, cognitive support, or environmental barriers? These approaches would not introduce new guidance or recommendations, but it may provide research teams with clearer points of entry when designing inclusive clinical trials for people with disabilities.

We thank the reviewer for this comment. We have created a more check-list style table by clinical trial phase to help make these recommendations more actionable. The table can be found on (Pages 20 to 21) titled **Table 2: Recommended Actions for Creating Disability Inclusive Clinical Trials**

The following lines were added to Methods and Results section on

> (Page 5, Lines 170 to 172)
>
> “The extracted data were analysed, summarized **(Supplemental Table 4-10)**, and used to develop recommendations and actions for disability inclusion in clinical trials.

> (Page 8, Lines 288 to 292)
>
> “To ensure that the evidence we have gathered is informative to facilitate disability-inclusive changes across different stages of clinical trials, we have transformed the findings into action-oriented recommendations that will help researchers design and implement inclusive trials. Actionable recommendations from each of the eight domains were identified and summarized in **Table 2** to improve the inclusion of people with disability throughout clinical trial stages.”

Another critique is that the methods do not mention how you developed the search strategy, e.g., what was your review question, and what search term framework did you use (PCC, etc.)? The inclusion criteria is also not clearly stated.

Thank you for your comment. We expanded the ‘Search Strategy’ section to clarify the search framework used (PCC) and include our research aim. The revised section is below:

> (Page 4, Lines 116 to 122)
>
> “A search strategy was developed with consultation with a librarian to address our research aim of identifying policies and practices of disability inclusion and exclusion in clinical trials. We followed the Population/Concept/Context (PCC) framework to develop a search query for each search concept using a combination of MeSH and keyword terms **(Supplemental Tables 1-2)**. Our population of interest was people with disabilities, the concept was disability inclusion and exclusion policies, practices, and guidance and the context for this review was clinical research.”

We also expanded the inclusion/exclusion criteria and separated it into its own ‘Inclusion/Exclusion subsection in ‘Methods’.

> (Page 4, Lines 128 to 146)
> “Inclusion/exclusion Criteria
> Publications from January 2019 to April 2025 were included in this review. We only included publications in English, and did not restrict documents based on disability type or location of publication. This project was a subset of a larger rapid scoping review focused on identifying guidance on the inclusion of people with disabilities in biomedical, health services, and public health research. We included written documents providing data on policies, recommendations, guidance, and practices of disability inclusion and exclusion in clinical research. Documents were not limited to a specific definition of disability to capture an overview of current clinical guidance across diverse disability definitions and types. Advanced Google and Google Scholar searches were used to identify publicly available information and documents including, but not limited to, press notes, organizational briefs, blog posts, institutional reports, guidelines, book chapters, and workshop notes which focused on disability inclusion/exclusion in clinical research.
>
> We excluded audio/visual content such as webinars and interviews; however, if a webinar or interview provided a written transcript, we included the transcript. Any documents not published in English or published prior to 2019 were excluded. Publications which did not provide guidance or policies on disability inclusion/exclusion in clinical research were excluded from this review.”

Also, please note that the PRISMA framework is a reporting guideline, it does not provide guidance on how to do a scoping review. Please correct the phrasing in the paper.

Thank you for pointing this out. We have corrected the phrasing to refer to the PRISMA-ScR framework as (Page 1, Lines 11 to 12; Page 4, Line 111) “reported according to PRISMA-ScR” or (Page 2, Line 56; Page 10, Line 363) “reporting according to PRISMA-ScR”

If the above can be addressed, this work would be an incredibly valuable resource.

VERSION 2 - REVIEW

**Reviewer**	**2**
**Name**	**Anil, Krithika**
**Affiliation**	**University of Plymouth**
**Date**	**14-Jan-2026**
**COI**	

Thanks to the authors for their work on addressing reviewer comments.

Table 2 seems to be a translation of Table 1’s “Summary” column. For clarity, please add a sentence or two explaining this, otherwise the two tables seem repetitive.

I have no further comments, I believe that this manuscript can be published once the above is addressed. I do not need to review this again.

Well done to the authors.

VERSION 2 - AUTHOR RESPONSE

Reviewer: 2
Dr. Krithika Anil, University of Plymouth
Comments to the Author:

Thanks to the authors for their work on addressing reviewer comments.

Table 2 seems to be a translation of Table 1's "Summary" column. For clarity, please add a sentence or two explaining this, otherwise the two tables seem repetitive.

I have no further comments, I believe that this manuscript can be published once the above is addressed. I do not need to review this again.

Well done to the authors.

We thank the reviewer for this comment. We have clarified the purpose of Table 2 in the last paragraph of the Results section:

(Page 8, Lines 288 to 291)
"To ensure that the evidence we have gathered is informative to facilitate disability-inclusive changes across different stages of clinical trials, we used the summary of guidance from each of the eight domains of recommendations **(Table 1)** to develop action-oriented recommendations organized by the clinical trial stages in **Table 2."**

Reviewer: 2
If you have selected 'Yes' above, please provide details of any competing interests.: N/A
